# Genetic and clinical predictors of CD4 lymphocyte recovery during suppressive antiretroviral therapy: Whole exome sequencing and antiretroviral therapy response phenotypes

**DOI:** 10.1371/journal.pone.0219201

**Published:** 2019-08-15

**Authors:** Ruth Greenblatt, Peter Bacchetti, Ross Boylan, Kord Kober, Gayle Springer, Kathryn Anastos, Michael Busch, Mardge Cohen, Seble Kassaye, Deborah Gustafson, Bradley Aouizerat, on behalf of the Women’s Interagency HIV Study

**Affiliations:** 1 UCSF School of Pharmacy, Department of Clinical Pharmacy, San Francisco, CA, United States of America; 2 UCSF School of Medicine, Department of Epidemiology and Biostatistics, San Francisco, CA, United States of America; 3 UCSF School of Medicine, Department of Medicine, San Francisco, CA, United States of America; 4 UCSF School of Nursing, Department of Physiological Nursing, San Francisco, CA, United States of America; 5 Johns Hopkins Bloomberg School of Public Health, Department of Epidemiology, Baltimore, MD, United States of America; 6 Albert Einstein College of Medicine and Montefiore Health Systems, Bronx, NY, United States of America; 7 Blood Systems Research Institute, San Francisco, CA, United States of America; 8 Stroger Hospital, Chicago, IL, United States of America; 9 Georgetown University Medical Center, Department of Medicine, Washington, DC, United States of America; 10 State University of New York, Downstate Medical Center, Department of Neurology, Brooklyn, NY, United States of America; 11 New York University School of Dentistry and Bluestone Center for Clinical Research, NY, NY, United States of America; University of Pittsburgh Centre for Vaccine Research, UNITED STATES

## Abstract

Increase of peripheral blood CD4 lymphocyte counts is a key goal of combined antiretroviral therapy (cART); most, but not all, recipients respond adequately and promptly. A small number of studies have examined specific genetic factors associated with the extent of CD4 recovery. We report a genome-wide examination of factors that predict CD4 recovery in HIV-infected women. We identified women in in a cohort study who were on cART with viral load below 400 copies, and drew racially and ethnically matched samples of those with good CD4 response over 2 years or poor response. We analyzed the exomes of those women employing next generation sequencing for genes associated with CD4 recovery after controlling for non-genetic factors identified through forward stepwise selection as important. We studied 48 women with good CD4 recovery and 42 with poor CD4 recovery during virologically-suppressive cART. Stepwise logistic regression selected only age as a statistically significant (p<0.05) non-genetic predictor of response type (each additional year of age reduced the odds of good recovery by 11% (OR = 0.89, CI = 0.84–0.96, p = 0.0009). After adjustment for age and genomic estimates of race and ethnicity, 41 genes harbored variations associated with CD4 recovery group (p≤0.001); 5 of these have been previously reported to be associated with HIV infection, 4 genes would likely influence CD4 homeostasis, and 13 genes either had known functions or were members of product families that had functions for which interactions with HIV or effects on lymphocyte homeostasis were biologically plausible. Greater age was the strongest acquired factor that predicted poor CD4 cell recovery. Sequence variations spanning 41 genes were independently predictive of CD4 recovery. Many of these genes have functions that impact the cell cycle, apoptosis, lymphocyte migration, or have known interactions with HIV. These findings may help inform new hypotheses related to responses to HIV therapy and CD4 lymphocyte homeostasis.

## Introduction

Numeric recovery of CD4 T cells (referred to as CD4 cells from here on) in peripheral blood is an important indicator of response to combination antiretroviral treatment (cART). The number of CD4 cells predicts the occurrence of HIV-associated opportunistic infections and cancers.[1–5] Suppression of HIV replication is a major determinant of immunologic recovery during cART. However, even when viremia is optimally controlled, 15–30% of cART recipients have very slow or minimal gains in circulating CD4 cells.[6–8]. Persons with poor CD4 cell recovery after cART-mediated suppression of HIV replication (so-called immunologic non-responders or INRs) may experience continued immune impairment and an increased risk of clinical complications [9–11].

The definition of INR after cART suppression varies in terms of time period and number of cells that is attained. Peripheral blood CD4 cell counts may increase for more than 5 years after initiation of suppressive therapy.[12] However, a CD4 cell gain of 50–150 cells/mm^3^ in the first year of suppressive therapy is a common standard for a good response, as are subsequent increases of 50–100 cells/mm3 per year. A short term gain followed by a sustained increase defines a treatment response that is considered adequate in the current Guidelines for use of Antiretroviral Agents.[13] Other definitions of CD4 cell count responses to treatment are based on expansion from pre-treatment CD4 counts and/or functional recovery. The varied definitions of immunologic recovery influence the reported proportion of optimal and INR cases; studies applying the most stringent recovery definition tend to report a larger proportion of suboptimal responses.

Poor CD4 cell recovery has been associated with age and telomere length, male sex, hepatitis C co-infection, pre-treatment CD4 counts, pre-treatment HIV RNA viral load (VL), specifics of the cART regimen, and duration of infection prior to cART initiation.[7, 10, 11, 14–20]

Previous studies demonstrated that host genetics influence expansion of CD4 populations during cART. The AIDS Clinical Trials Group examined 137 single nucleotide polymorphisms (SNPs) among 17 genes and found that SNPs of *TNFSF10*, *TNF*, *BCL2L11*, *IL15RA*, and *IL15* were associated with differential CD4 cell recovery at 12 months of cART virologic suppression.[21] *IL7R* gene polymorphisms were associated with rate of CD4 cell recovery in a large cohort of cART recipients, a finding consistent with the known role of IL-7 in establishing CD4 lymphocyte homeostasis.[22] *CCR5* genotype/*CCL3L1* copy number was associated with extent and rate of CD4 cell recovery in another cohort.[23] cART recipients who were homozygous for HLA-Bw4 were significantly more likely to be INRs than other individuals.[24, 25] Mitochondrial DNA genotype also influences rate and extent of CD4 cell recovery among cART recipients.[26, 27] The participants of these studies were either predominantly male or the proportion of females was not specified; determination of factors associated with CD4 lymphocyte recovery in females is important because sex dimorphisms are recognized for immune responses, circulating numbers of T cells, and intensity of HIV viremia. In the study of *IL-7R* haplotype 2 among Ugandan cART recipients[25]; female sex was associated with significantly longer time until reaching a threshold value of 500 cells/mm3, but this association was not independent of other factors such as pre-treatment CD4 cell count and age.

We report herein the associations of demographic and clinical characteristics and exome sequence variations with CD4 cell recovery of women (participants of the Women’s Interagency HIV Study, WIHS), limited to women who experienced rapid expansion of peripheral blood CD4 cell count or women who had slow or minimal expansion of these cells while receiving cART with suppression of plasma VL to below 40 copies/ml.

## Materials and methods

### Study population

WIHS is a longitudinal, multisite, observational cohort study of HIV infection among U.S. women.[28, 29] Use of cART was defined according to expert HIV treatment guidelines.[13] Our analysis used a dichotomized measure of self-reported adherence of the proportion of doses of antiretroviral medications taken as being either ≥95% or <95% of what the provider instructed over the last six months. Peripheral blood CD4 counts and VL quantitation in plasma were determined via standard assays in laboratories that participated in the NIAID laboratory quality assurance program. The CD4 nadir is defined as the lowest point to which the CD4 count has dropped and is predictive of slower immune recovery and long-term morbidity. Nadir CD4 count was the lowest value measured by WIHS, or pre-WIHS ascertained via medical record review. Anti-Müllerian hormone (AMH) levels in plasma were measured using a commercially available ELISA with a lower limit of detection of 0.08 ng/ml. AMH is an indicator of ovarian follicular reserve, and falls below assay detection 3–5 years prior to menopause, a time that corresponds to the onset of consistent estradiol depletion. [30] Informed consent was provided by all participants via protocols approved by institutional review committees or boards (IRB) at each affiliated institution with consent to studies of host genetics was specifically obtained for women who contributed to this study. The IRBs at the Chicago site were Cook County CORE Center IRB, Cook County Health & Hospitals System IRB, Northwestern University IRB, Northwestern Memorial Hospital IRB, Rush University IRB, Rush-Presbyterian-St. Lukes IRB, University of Illinois at Chicago IRB. The IRBs at the Los Angeles/Southern California site were University of California at Los Angeles Medical Center IRB, Western IRB, University of Hawaii/Kapi’olani Health Research Institute IRB, University of Southern California IRB. The IRB at the New York Brooklyn site was SUNY Downstate Medical Center Institutional Review Board. The IRBs at the New York Bronx site were Albert Einstein College of Medicine IRB, Beth Israel IRB, Mt. Sinai Medical Center IRB. The IRBs at the San Francisco Bay Area site were Alameda Health System IRB, Sutter Health IRB, University of California, San Francisco IRB, California Committee for the Protection of Human Subjects (CPHS). The IRBs at the Washington District of Columbia site were Georgetown University Medical Center IRB, Howard University IRB, Inova Health Systems IRB, Montgomery County Health Department IRB, Whitman Walker Clinic IRB.

### Phenotype determination

Study visits during reported cART use were categorized according to whether VL was suppressed and according to whether CD4 counts demonstrated adequate gains over pretreatment and nadir values. Imputed values for CD4 counts or VL were applied to one or two visits with missing values for these measures if there was no change in cART status between at least two nearby visits with the measurements available. To mitigate volatility in CD4 counts, non-missing CD4 counts were smoothed using values from two or three nearby visits, when available. Detailed specification of the phenotyping algorithm is provided in online **S1 Appendix**, and the SAS program implementing it is in online **S2 Appendix**. These appendices include programming for phenotypes that were not used for this study.

### Selection for this study

**Fig 1:**
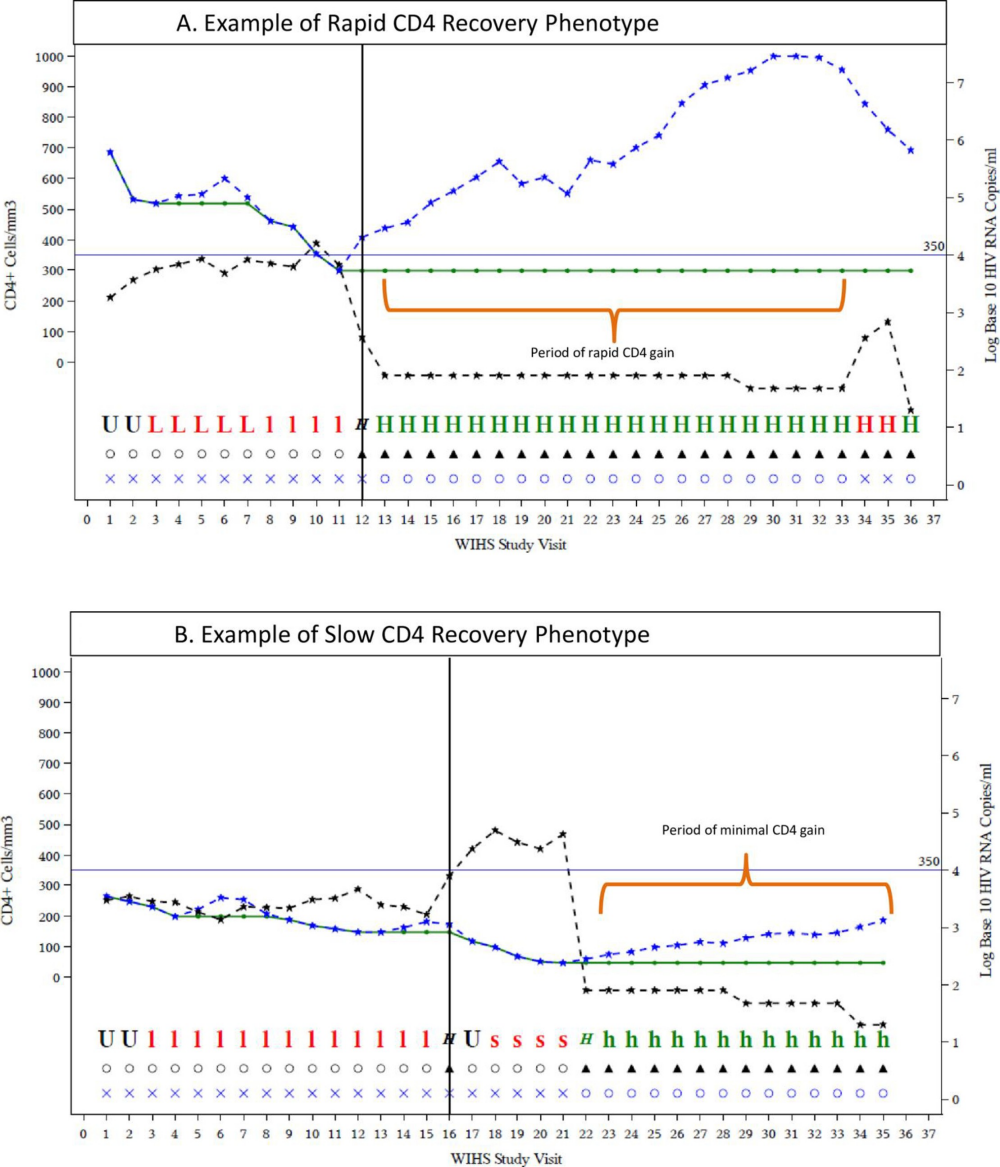
**Example plots of CD4 and VL changes overtime for a good CD4 responder (1A) and a poor responder (1B)**. Fig 1A legend. WIHS Study Visit denotes visit number (2 visits per year). The vertical black line at visit 16 denotes the timepoint at which the beginning of the phenotypic window begins, which is followed by a period of rapid CD4 T cell gain (orange bracket). The following graphics were used to display relevant clinical, disease, and treatment information: dashed blue line denotes CD4 T cell count, solid green line denotes nadir CD4 T cell count, dashed black line denotes HIV viral load, HIV antiretroviral therapy (ART) status was denoted using ◯ (no ART) or ▲ (combination ART (cART) visit, and HIV viremia status was denoted using ◯ designated in blue font (HIV RNA below the assay detection limit) or ✖ designated in blue font (HIV RNA greater than or equal to the assay detection limit. The visit phenotype was denoted using the following symbols: **U** (not determined due to the lack of sufficient observation), designated in red font (long term off cART and viremic with stable CD4), **l** designated in green font (long term off cART and viremic with declining CD4), ***H*** (first cART visit), **H** designated in red font (on cART with viremia), or **H** designated in green font (on cART with virologic suppression). Fig 1B legend. WIHS Study Visit denotes visit number (2 visits per year). The vertical black line a visit 12 denotes the timepoint which is followed by a period of minimal CD4 T cell gain (orange bracket). The graphics were used to display relevant clinical, disease, and treatment information are the same as for Fig 1A, except that the visit phenotype had the following additional symbols: **s** designated in red font (short term off cART with viremia and declining CD4), ***H*** designated in green font (reinitiated cART visit), or **h** designated in green font (virologic suppression with minimal CD4 gain).

Initial selection for this study was completed using the phenotyping detailed in the preceding section, and was based on the initiation of cART, having plasma VL below assay detection (e.g., <80 copies) for ≥1 year, and having either the most rapid and durable gains in CD4 counts for at least 2 years or the poorest gains for at least one year during cART visits with viral suppression. Because race is associated with total leukocyte counts in peripheral blood, we matched the good and poor responder groups by race and ethnicity. Our goal was to select 50 women with rapid CD4 cell responses and 50 with slow CD4 cell responses, matched for race and ethnicity. Initial screening with the algorithm (online S1 appendix) identified 201 rapid responders and 78 slow responders. We reviewed longitudinal plots of viral load, CD4 cell count, and treatment status (examples in **Fig 1**) stratified by race (i.e., African-American, White) and Hispanic ethnicity, and identified 46 clearly slow responders. We identified 50 race-matched rapid responders with similarly clear and rapid CD4 cell expansion after initiation of suppressive cART.

Ninety-two of the 96 initially selected women had available samples for exome sequencing (examples provided in **Fig 1**). Two additional eliminations occurred following genetic data collection; one had an inadequate genomic material, and exome sequencing failed in the second, resulting in a final sample of 90 women.

### Statistical analyses

We used forward stepwise logistic regression to select variables from a set of demographic, clinical, and treatment variables, for inclusion in a final model of acquired factors associated with good recovery. Predictors were required to have a p-value for association with CD4 response group of <0.05 in a multivariate regression. SAS version 9.4 was used for these analyses.

#### Whole exome sequencing

Banked peripheral blood mononuclear cells underwent genomic DNA extraction using the Puregene DNA Isolation System (Invitrogen, Carlsbad, CA), quantitated, and normalized to a concentration of 45 ng/mL. Approximately 6.75 micrograms of DNA was subjected to sequencing at an average depth of read of 50X. Sequencing was performed by Expression Analysis Inc. (Durham, NC). Prior to library construction and sequencing, DNA was evaluated for evidence of degradation by electrophoresis.

Exome sequencing was completed with 100 base-pair paired-end sequencing. A PhiX control sample was included on all flow cells. DNA fragments were amplified using Clonal Single Molecule Array technology and Sequencing-by-Synthesis using Reversible Terminator Chemistry. Sequence data was stored as FASTQ files for each specimen. Sequences were required to pass Illumina (San Diego, CA) purity filter reads and was accompanied by a per-base quality score, as defined by Illumina-scaled Phred metric.

#### Variant calling file generation procedure

Paired-end whole-exome reads were processed and aligned to the human genome. The alignments were used to call variants in order to create the Variant Calling Format (VCF) file using the Genome Analysis Toolkit from The Broad Institute (GATK) following best practices.[31, 32] A diagram of the VCF file generation procedure is provided in **S1 Fig**. In brief, paired-end reads were clipped from adapters using fastq-mcf from the EA-utils package (https://code.google.com/p/ea-utils/). Paired-end libraries were evaluated for quality using FastQC (http://www.bioinformatics.babraham.ac.uk/projects/fastqc/). Paired-end reads were aligned to the reference genome assembly GRCh37/hg19. Alternate loci and patches were excluded. Alignments were performed with bwa-mem (http://bio-bwa.sourceforge.net).[33] Variant calling was performed using GATK version 3.4–46 (https://www.broadinstitute.org/gatk).

Alignments were processed with samtools (http://www.htslib.org)[34] to remove PCR duplicates. Read Groups lines were defined in the BAM file using AddOrReplaceReadGroups in picard-tools (http://picard.sourceforge.net). Re-alignment was performed in regions around known indels with the RealignerTargetCreator and IndelRealigner in GATK using the gold standard indels from Mills, the 1000 Genomes Project (KGP), and the KGP phase 1 indels (GATK Resource Bundle, version 2.8, ftp.broadinstitute.org). Recalibration of the base quality scores was performed to assign an empirically accurate error model to the bases with BaseRecalibrator/PrintReads in GATK using the known variation from the gold standard indels from in the above listed sources and dbSNP 138. Variant calling was performed per sample with the GATK HaplotypeCaller in GCVF mode. All samples were called together to gain more confidence in weak calls with GenotypeGVCFs and dbSNP 138. Variants were recalibrated using VariantRecalibrator using known variation data from dbSNP 137, HapMap 3.3, and KGP Omni 2.5. A Variant Quality Score logarithm of odds was calculated for each variant using ApplyRecalibration (filer level = 99.0). Final annotations for variant filtration were performed with SelectVariants for approximate read depth (DP<8) and genotype quality (GQ<20) as previously suggested.[35] Only SNP variation was called. Genomic data for the WIHS is indexed in the database of Genotypes and Phenotypes (dbGaP); the accession number is phs001503.

#### Genetic association analyses

Allele and genotype frequencies were determined by gene counting. Population substructure in good and poor CD4 response groups was estimated by principal component analysis (PCA). The first two principal components (PCs) were sufficient to estimate the substructure of the cohort due primarily to genetic racial and ethnic differences, which were employed to control for the potential confound of ancestry-based substructure in the multiple regression analyses.

The approaches used for association analysis in host genomic studies typically focus on common polymorphisms but are poorly suited to detect the impact of rare polymorphisms and cannot estimate their combined effect or “variant burden”. In order to leverage the availability of common *and* rare polymorphisms yielded from whole exome sequencing, two approaches to estimate the magnitude of variant burden with the CD4 recovery phenotype were considered: the Combined Multivariate and Collapsing (CMC) method[36] and the Kernel-Based Adaptive Collapsing (KBAC) method[37] Both methods collapse variants into a single covariate based on gene regions (i.e., a gene). CMC can detect the effects of both common and rare alleles, while KBAC detects effects of rare alleles only. Gene associations identified by both CMC and KBAC would suggest that both common and rare variants contribute to the observed association, while gene associations identified by CMC or KBAC alone, would suggest that the variant burden at said gene was due primarily to common or rare variants, respectively. Both unadjusted and adjusted (i.e., age, genomic estimates of population substructure) associations were calculated. For CMC and KBAC, gene regions were defined by RefSeq release 63. Statistical analyses were carried out using HelixTree software (Golden Helix, Bozeman, MT).

The CMC method groups variants according to the frequency of each polymorphism within a gene region to perform a multivariate test collapsed over the gene region to estimate the association with the phenotype. Both common (i.e., ≥5% minor allele frequency [MAF]) and rare (i.e., <5% MAF) SNPs are included in CMC analyses. In the current study we defined five potential groups, or bins, based on the frequency of the rare allele for each SNP as provided by KGP: <1%, 1%≤x<2.5%, 2.5%≤x<5%, 5%≤x<10%, ≥10%. SNPs identified by whole exome sequencing in WIHS not present in the KGP reference database were assigned to the bin defined as <1% because the variant was assumed to be too rare to be detected in KGP.

The KBAC method collapses the genotypic information across all polymorphisms in a gene region into compound, multi-marker genotype without the need for binning. However, common SNPs (i.e., ≥5% MAF) must be excluded from analysis. The counts of the multi-marker genotypes are used to perform a multivariate test to estimate their association with the outcome. The KBAC test weights multi-marker genotypes based on risk for the outcome with increased risk genotypes assigned higher weights to better distinguish between causal and non-causal genotypes. Accordingly, the KBAC test is a one-sided test.

Initially, both CMC and KBAC regression analyses were performed at the level of the gene with all transcripts consolidated into a single region. In order to evaluate the possibility that associations may be narrowed to specific transcripts, the CMC and KBAC regression analyses were repeated for every transcript only for those gene regions that met *a priori* significance thresholds to minimize the added multiple testing penalty. For candidate gene analyses, a significance threshold of p<0.05 was selected *a priori* for the evaluation of previously identified candidate genes for CD4 recovery. Based on the exploratory nature of the exome analyses using both CMC and KBAC covariate-adjusted models, we report here the genetic predictor terms that had a gene-wise permutation-based p<0.001 by either CMC or KBAC after one million permutations, a threshold that is more appropriate when conducting gene-wise association studies.[38] The CMC analyses evaluated 18,987 genes while KBAC analyses evaluated 19,653 genes; genes associated with CD4 recovery using either method where retained.

Genes that were associated with CD4 recovery group were crossed referenced via the HIV:host protein interaction database (https://www.ncbi.nlm.nih.gov/genome/viruses/retroviruses/hiv-1/interactions/). Both direct (i.e., direct host protein:viral protein) and indirect (HIV protein interaction with a host protein that interacts with the protein encoded for by an identified CD4 recovery gene) were considered.

## Results

### Clinical factors

Ninety WIHS participants contributed to analyses reported here; 42 met criteria for poor CD4 recovery on virologically-suppressive cART and 48 women demonstrated good CD4 expansion on virologically-suppressive cART. **Fig 1**provides examples of CD4 and VL plots for women demonstrating (Fig 1A) good (n = 48) and (Fig 1B) poor (n = 42) CD4 gain.

**Table 1**summarizes the characteristics of the two CD4 response groups (white columns), and the associations estimated by univariate logistic regression (gray columns). Even though we limited analysis to women who had HIV RNA levels below assay threshold during the CD4 response time period, ≤95% adherence to the prescribed regimen was common. CD4 nadir was associated with CD4 response phenotype (OR = 2.0 per 100 cell/ml, 95% CI 1.34–3.1, p = 0.0008). However, CD4 count and the amount of increase above CD4 nadir during the response period are used in the CD4 response group definition, so the meaning of this association is questionable.

**Table 1:** Characteristics of two outcome groups with univariate analysis.

Predictor of Rapid Response	CD4 RESPONSE GROUPS		RESULT	
	Good	Poor	OR for rapid response	p-value
	mean/std dev (n)	mean/std dev (n)	OR (95% CI)	
Age at start of response period	39.2 ± 7.5 (n = 48)	45.5 ± 8.2 (n = 42)	0.894 (0.838–0.955) per year	0.0009
CD4 T cell count nadir cells/ml	255.3 ± 189.4 (n = 48)	127.5 ± 81.8 (n = 42)	2.0 (1.34–3.1) per 100 cells/ml	0.0008
Maximum plasma HIV RNA before cART initiation (log_10_)	4.4 ± 0.9 (n = 48)	4.5 ± 0.9 (n = 42)	0.985 (0.616–1.574)	0.95
	**n (% of column)**	**n (% of column)**		
Self-Reported Race^◆^: White (non-Hispanic)	12 (25.0%)	12 (28.6%)		
White (Hispanic)	7 (14.6%)	5 (11.9%)		
African-American (non-Hispanic)	16 (33.3%)	12 (28.6%)		
Other	13 (27.1%)	13 (31.0%)		
AMH^▲^during phenotype	1 (2.1%)		0.221 (0.087–0.564)	
Not available	10 (20.8%)	2 (4.8%)		0.0016
Below detection (≤0.09ng/ml)		22 (52.4%)		
Clinical AIDS occurred prior to CD4 response phenotype	17 (35.4%)	24 (57.1%)	0.411 (0.176–0.963)	0.041
HCV RNA positive at the time of cART initiation	11 (22.9%)	18 (42.9%)	0.396 (0.160–0.984)	0.046
Reported receiving ddI or d4T prior to CD4 response phenotype	32 (66.7%)	36 (85.7%)	0.333 (0.116–0.955)	0.041
Reported adherence to prescribed cART regimen during phenotype:			3.300 (1.288–8.5)	0.013
Not available	5 (10.4%)	2 (4.8%)		
>95%	33 (68.8%)	20 (47.6%)		
≤95%	10 (20.8%)	20 (47.6%)		
Was a tobacco smoker prior to start of response phenotype	15 (31.3%)	22 (52.4%)	0.413 (0.175–0.976)	0.044

Abbreviations: cART = combined HIV antiretroviral therapy; CI = confidence interval; HCV = hepatitis C virus; n = sample size; OR = Odds Ratio; std dev = standard deviation

◆ participants in the two outcome groups were matched by race

▲AMH = antiMüllerian hormone measured in plasma.

Age at the onset of the response-defining treatment period was strongly associated with CD4 response group (OR for a good response = 0.89 per year, 95% CI = 0.838–0.955, p = 0.0009). Being a tobacco smoker at start of the CD4 response phenotype was also associated with CD4 response group: OR for a good response = 0.413, 95% CI = 0.175–0.976, p = 0.044). Plasma AMH level was available for 87 women. Undetectable AMH was associated with poor response group: OR for a good response = 0.221, 95% CI = 0.087–0.564, p = 0.0016.

All women reported current or past use of nucleoside antiretroviral drugs, though prior use of ddI or d4T was associated with poor CD4 response (OR for a good response = 0.333, 95% CI = 0.116–0.955, p = 0.041). Self-reported adherence of >95% to the cART regimen was associated with a good CD4 response (OR = 3.300, 95% CI = 1.288–8.5, p = 0.013).

Most of these covariates are associated with each other. For current smokers age and years smoked will line up exactly, and even for former smokers there will likely be a relation. Because ovarian follicular reserve declines with age, AMH detection also declines with age. ddI and d4T were used earlier in the epidemic, and so will also be associated with age. To sort these out we constructed a multivariate model with forward stepwise logistic regression, with p<0.05 for inclusion. Nadir CD4 count was not included as a candidate predictor in the model because it is linked with criteria for group assignment. Age had the smallest p-value on its own, and none of the other variables had p<0.05 after controlling for age. The confidence intervals for many of those other predictors include substantial effects, so one should not conclude this finding to mean they don’t matter. It just means we don’t know much about how or if they matter.

However, our decision rule generates a final model of non-genetic factors that includes only age. Nadir CD4 count was not included as a candidate predictor in the model because it is linked with criteria for group assignment.

### Whole exome and candidate gene analyses

**Table 2** details the 41 genes that had p≤0.001 for predicting response group by CMC (a regression analysis approach that evaluates the genetic burden of both common and rare alleles) and/or KBAC (a regression analysis approach that evaluates the genetic burden of rare alleles), along with genes previously reported to be associated with HIV-related outcomes (i.e., candidate genes). The reported values are from regression analyses controlling for age (based on the multivariate analysis of non-genetic variables discussed immediately above) and the first two ancestry principal components that were included to account for any residual variability in population substructure due to race and/or ethnicity after matching on self-reported race and ethnicity. Five of these genes were previously reported to be associated with HIV-related outcomes, including *HECW2*, *CLCN3*, *ALG2*, *SRP14*, and *CCL25*. Another four genes have functions that would likely influence CD4 lymphocyte homeostasis; these were: *SLC12A2*, *USP12*, *GMIP*, *KLC3*. Eleven of the genes associated with CD4 response had unknown functions. The remaining 21 genes either had known functions or were members of product families that had functions for which interactions with HIV or effects on lymphocyte homeostasis were biologically plausible. None of the 8 candidate genes previously reported to be linked with HIV treatment responses were statistically significantly associated (p<0.05) with CD4 response group in this study. A detailed description of the estimates of association for both CMC and KBAC is provided in online **S1 Table** and the polymorphisms incorporated into each gene level burden regression analysis is provided in online **S2 Table**.

**Table 2:** Whole exome and candidate genes associated with CD4 response group.

Gene	Gene Name	Position	Function	CMC p-value^1^	KBAC p-value^1^	HIV protein interaction
**Whole Exome Design**						
*LCE1D*	Late cornified envelope 1D	1:152769227	Polymorphisms associated with eczema.[39]		0.00004	unknown
*YPEL5*	Yippee like 5	2:30369750	Codes for a protein, of a family involved in cell cycle progression and cellular proliferation.[40]		0.00014	*ADCYAP1*^*t*^, *CNRIP1*^*g*^, *COPS5*^*v*, *g*^, *CTSA*^*v*^, *DDX5*^*R*, *e*, *p*, *n*, *g*, *r*, *v*^, *EFHC2*^*R*^, *ELAVL1*^*e*, *p*, *n*, *g*^, *ESR2*^*e*, *t*^, *GPS2*^*V*^, *HIST1H3E*^*t*^, *HLA-DPB1*^*e*, *n*, *g*, *t*, *u*^, *IKBKE*^*e*^, *PRG2*^*t*^, *RAB8A*^*e*, *p*, *n*, *g*^, *RANBP9*^*p*^, *SYNCRIP*^*e*, *p*, *n*, *g*, *r*^
*HECW2*	HECT, C2 and WW domain containing E3 ubiquitin protein ligase 2	2:197063977	Family members associated with HIV enhancement and HIV-1 Gag protein ubiquitination.[41]	0.00057	0.58511	*ABO*^*R*^, *ACSL4*^*v*^, *ACTL6A*^*t*^, *AFF4*^*t*^, *AHCY*^*t*, *p*^, *ALDOC*^*v*^, *AMOT*^*g*^, *AMOTL1*^*g*^, *AMOTL2*^*g*^, *ANAPC1*^*p*^, *ANXA1*^*e*^, *ARF4*^*e*, *p*, *n*, *g*^, *ASXL2*^*R*^, *BAIAP2*^*R*, *e*, *g*^, *CALM3*^*e*, *n*, *g*^, *CAPZB*^*t*^, *CBX3*^*t*, *v*^, *CBX5*^*t*, *v*^, *CCNA1*^*t*, *V*^, *CCNK*^*n*^, *CDC16*^*R*^, *CDC20*^*t*^, *CDT1*^*v*^, *CENPF*^*v*^, *CFL1*^*e*, *n*, *g*, *t*^, *CHEK1*^*v*^, *CIT*^*R*, *g*^, *CKMT1B*^*p*^, *CPSF2*^*r*^, *CRTC2*^*R*^, *CSNK2A1*^*r*, *u*, *g*, *p*^, *CSTF3*^*r*^, *CTR9*^*R*^, *CYCS*^*R*, *e*, *n*, *t*, *v*, *p*^, *DDX24*^*r*^, *DHX36*^*r*^, *DNAJA2*^*R*, *V*^, *DNAJC8*^*p*^, *DRG1*^*e*, *p*, *n*, *g*^, *DYSF*^*R*, *v*^, *ECM1*^*t*^, *EEF1D*^*e*, *p*, *n*, *g*, *t*^, *EGFR*^*R*, *g*^, *EIF2AK2*^*e*, *n*, *g*, *t*, *V*, *u*^, *EIF3H*^*R*, *p*^, *EIF3I*^*p*, *r*^, *ELAVL1*^*e*, *p*, *n*, *g*^, *EMG1*^*r*^, *EP400*^*t*^, *ETF1*^*R*, *p*, *g*^, *EXOSC7*^*r*^, *FRG1*^*g*, *t*^, *GRN*^*t*^, *GSK3B*^*e*, *n*, *t*, *v*, *u*^, *GTF2F2*^*e*, *t*^, *HACD3*^*e*, *p*, *n*, *g*^, *HSD17B12*^*u*^, *HSD17B4*^*e*, *p*, *n*, *g*, *v*^, *HSPH1*^*e*^, *INCENP*^*v*^, *KAT2A*^*R*, *t*, *p*^, *KAT8*^*p*^, *KDM4D*^*R*^, *KMT2A*^*p*^, *KPNA1*^*R*, *r*, *v*, *p*, *g*^, *LAMB1*^*t*^, *LAMC1*^*t*^, *LITAF*^*V*^, *LMNB1*^*e*, *t*, *v*^, *LSM14B*^*R*^, *MAP1S*^*R*, *g*^, *MAP2*^*e*, *t*, *p*^, *MARK1*^*R*^, *MECP2*^*n*^, *MED14*^*R*, *t*^, *MOS*^*R*^, *MPG*^*r*^, *MRPS9*^*r*^, *MTHFD1L*^*V*^, *NELFA*^*t*^, *NPLOC4*^*u*^, *OSBPL8*^*e*^, *PAK4*^*R*^, *PBRM1*^*t*^, *PHF3*^*R*^, *PIP5K1A*^*e*^, *PKN2*^*R*, *g*, *u*^, *POLR2B*^*e*, *t*, *V*, *v*^, *PPIB*^*R*, *e*, *r*, *g*^, *PRDX2*^*p*^, *PRKG1*^*t*^, *PSMA7*^*R*, *t*, *V*, *p*^, *PSMB1*^*t*, *V*, *p*^, *PSMB3*^*t*, *V*, *p*^, *PSMB7*^*t*, *V*, *p*^, *RAD21*^*R*, *v*^, *RBBP6*^*g*^, *RBM15B*^*g*, *p*^, *RBM28*^*p*^, *RBM42*^*r*^, *RNF2*^*t*^, *RPL24*^*e*, *p*, *n*, *g*^, *SEH1L*^*e*^, *SENP3*^*e*^, *SF3A2*^*r*^, *SMAD5*^*t*^, *SNRPB2*^*t*^, *SNW1*^*R*, *t*, *p*^, *SP2*^*t*^, *SPAG5*^*e*^, *SPAST*^*R*^, *SRBD1*^*r*^, *SRSF10*^*e*, *p*, *n*, *g*, *t*^, *TECR*^*R*^, *TFAP2A*^*e*, *n*^, *TP53*^*R*, *e*, *n*, *g*, *r*, *t*, *V*, *v*, *u*, *p*^, *TP73*^*t*^, *TRIM25*^*R*, *g*^, *TUBG1*^*e*, *g*, *t*, *v*^, *UBE2D2*^*V*, *p*^, *UBE2L3*^*R*, *n*^, *UPF3B*^*R*, *e*, *p*, *n*, *g*^, *WDR36*^*r*^, *XRN1*^*g*^, *YTHDF3*^*g*^, *ZC3H7B*^*R*^, *ZCCHC17*^*R*^, *ZMYND8*^*e*^, *ZNF579*^*t*^
*CHDH*	Choline dehydrogenase	3:53850324	Polymorphisms associated with a variety of developmental phenotypes and some cancers.	0.00085	0.10891	*MAP1LC3A*^*e*, *n*, *g*, *t*, *V*, *p*^, *NDUFS7*^*R*^, *NOTCH2NLA*^*R*^, *RPL8*^*e*, *p*, *n*, *g*^, *SQSTM1*^*R*, *e*, *n*, *t*, *V*, *g*^, *GGA1*^*n*, *g*, *v*^, *HSPB1*^*V*, *v*^, *LARP7*^*t*^, *LGALS3*^*R*, *g*, *r*, *t*, *v*^, *LGALS8*^*R*^, *SLC9A3R1*^*e*, *p*^, *TRIM25*^*R*, *g*^
*SLITRK3*	SLIT and NTRK-like family member 3	3:164904508	Codes for a transmembrane protein, the expression of which is a related to the biology of some tumors and behavioral phenotypes.[42, 43]	0.00051	0.77778	*EGFR*^*R*, *g*^, *HNRNPL*^*p*^, *KDM5B*^*R*^
*DCAF4L1*	DDB1 and CUL4-associated factor-4-like 1	4:41983713	Codes for a protein of unknown function		0.00058	*COPS5*^*v*, *g*^, *COPS6*^*v*^, *CUL4A*^*r*, *V*, *v*^, *CUL4B*^*r*, *v*^
*CLCN3*	Chloride voltage-gated channel 3	4:170541672	Abnormalities of ion channel functions hypothesized to influence HIV associated dementia.[44]	0.00085	0.02083	*GGA1*^*n*, *g*, *v*^, *HSPB1*^*V*, *v*^, *LARP7*^*t*^, *LGALS3*^*R*, *g*, *r*, *t*, *v*^, *LGALS8*^*R*^, *SLC9A3R1*^*e*, *p*^, *TRIM25*^*R*, *g*^
*ARRDC3-AS1*	ARRDC3 antisense RNA 1	5:90676164	Gene of unknown function.		0.00003	unknown
*SLC12A2*	Solute carrier family-12 member 2	5:127419483	Member of a pathway that regulates T cell adhesion and migration.[45] Polymorphisms associated with CNS functional variants.[46, 47]	0.00016	1.00000	*BMPR1A*^*e*, *n*, *t*^, *C1QBP*^*e*, *r*, *t*, *v*^, *CCDC8*^*R*, *g*^, *CDH1*^*e*, *t*, *v*, *u*^, *EGFR*^*R*, *g*^, *ESR2*^*e*, *t*^, *HRAS*^*e*, *n*, *t*^, *KRAS*^*e*, *t*^, *LGALS3*^*R*, *g*, *r*, *t*, *v*^, *LGALS8*^*R*^, *MAPK14*^*e*, *n*, *t*, *v*^, *NRAS*^*R*, *e*, *n*, *t*^, *NTRK1*^*e*, *v*^, *SEC13*^*e*^
*SLC25A27*	Solute carrier family-25 member 27, or mitochondrial uncoupling protein 4	6:46620652	In family of genes linked to renal toxicity of tenofovir.[48] Polymorphisms linked to schizophrenia.[49, 50]	0.00049	0.71429	R
*TCF21*	Transcription factor 21	6:134210259	Codes for a protein transcription factor expressed in some mesenchymal and epithelial tissues. Polymorphisms of this gene are associated with several cancers and vascular disease.[51–54]	0.00048	1.00000	*APEX1*^*R*, *r*^, *GTF3C5*^*t*^, *TCF12*^*R*, *v*^, *TCF3*^*t*^
*MIR3662*	microRNA 3662	6:135300476	The expression of many microRNAs differs by HIV status and disease characteristics and polymorphisms of microRNA genes is known to influence the outcomes of several viral infections.[55, 56]		0.00003	unknown
*VTA1*	Vesicle trafficking 1	6:142468299	Involved with cellular vesicular escort processes that enable viral budding.[57, 58] Polymorphisms associated with characteristic of microcirculation.[59]	0.00073	0.25134	*APP*^*e*, *t*, *p*^, *CDK4*^*t*^, *CGAS*^*g*, *p*^, *CHMP2A*^*R*, *g*^, *CHMP3*^*e*, *g*^, *CHMP5*^*R*^, *CTTN*^*g*^, *DARS*^*e*, *p*, *n*, *g*^, *DLD*^*p*^, *ELAVL1*^*e*, *p*, *n*, *g*^, *GSK3A*^*n*^, *GSK3B*^*e*, *n*, *t*, *v*, *u*^, *KCTD13*^*p*^, *LYST*^*n*^, *MAPK3*^*e*, *n*, *r*, *t*, *V*, *v*, *g*, *p*^, *NF2*^*R*, *n*^, *NTRK1*^*e*, *v*^, *RABAC1*^*e*^, *SPAST*^*R*^, *VCAM1*^*R*, *e*, *t*, *u*^, *VPS4A*^*R*, *n*, *g*, *u*^, *VPS4B*^*n*, *g*, *u*^, *ZBTB16*^*V*^, *ZNRD2*^*p*^
*GRHL2*	Grainyhead-like transcription factor 2	8:102504668	A transcription factor that influences tissue development and is associated with several cancers. Polymorphisms linked to auditory phenotypes.[60]	0.00065	1.00000	*APP*^*e*, *t*, *p*^, *ESR2*^*e*, *t*^, *HNRNPL*^*p*^
*NUDT2*	Nudix hydrolase 2	9: 34329504	Member of nucleotide pyrophosphatase family the expression of which is linked with HIV progression phenotype.[61]		0.00003	*MCM6*^g-p,t,V^
*ALG2*	Alpha-1,3/1,6-mannosyltransferase	9:101978707	May interact with HIV 1 enhancer binding protein 3.[62]		0.00108	*CD79B*^*e*^, *HMOX2*^*e*^, *HSP90AA1*^*p*, *n*, *g*, *t*, *V*^, *LAMP1*^*e*, *n*, *g*, *t*, *v*, *u*^, *PDCD6IP*^*R*, *e*, *n*, *g*^, *PTK2B*^*R*, *e*, *n*, *t*, *p*^, *PTPN23*^*g*, *t*^, *SEC31A*^*e*^, *SRI*^*g*^, *TGOLN2*^*g*, *u*^, *TSG101*^*R*, *n*, *g*, *p*, *v*, *u*^, *VPS28*^*g*^, *VPS37A*^*g*^
*CARD9*	Caspase recruitment domain family member 9	9:139258408	Regulatory role in cellular apoptosis and a participant of monocyte signaling, linked with autoimmunity.[63, 64]	0.00065	1.00000	*AMOTL2*^*g*^, *AXIN1*^*R*, *t*^, *CDCA7L*^*t*, *p*^, *CSNK2A1*^*r*, *u*, *g*, *p*^, *DAXX*^*p*^, *KIAA0408*^*R*^, *KIAA0408*^*R*^, *MFAP1*^*r*^, *SUMO1*^*g*, *p*^, *TLE5*^*R*^, *TRIM29*^*R*^, *TRIM42*^*R*^, *TRIM62*^*g*^, *VPS28*^*g*^, *ZNF587*^*R*^, *ZNF688*^*R*^
*RP11-383C5*.*3*	Uncharacterized protein	10:127371812			0.000016	unknown
*CTAGE7P*	CTAGE family member 7, pseudogene	10:131904273			0.00014	unknown
*MRVI1-AS1*	Murine retrovirus integration site homolog—antisense RNA	11:10562783		0.00100	0.56383	unknown
*QSER1*	Glutamine and serine rich 1	11:32914792		0.00044	0.50000	*AAK1*^*R*, *n*^, *BRD1*^*R*^, *CSK*^*e*, *t*^, *ESR2*^*e*, *t*^, *FHL3*^*R*^, *FOXJ2*^*R*^, *HNRNPH2*^*e*, *p*, *n*, *g*, *r*^, *HNRNPL*^*p*^, *PCGF1*^*R*^, *PHF12*^*R*^, *PLEC*^*V*, *v*^, *UBE2D2*^*V*, *p*^
*KCTD14*	Potassium channel tetramerization-domain-containing 14	11: 77726761		0.00024	0.05157	unknown
*SNORA2C*	Small nucleolar RNA, H/ACA box 2c	12:49048165	This is a recently annotated gene that is predicted to encode a non-coding RNA.		0.00003	unknown
*USP12*	Ubiquitin specific peptidase 12	13:27640287	Recently reported to influence the T-cell receptor complex during cell surface signaling [65] and regulates lymphoblastoid cell growth.[66]	0.00017	0.40000	*APP*^*e*, *t*, *p*^, *CBLB*^*e*^, *DHX8*^*R*^, *GORASP1*^*n*^, *GRB2*^*n*, *p*, *t*^, *HIST2H2AC*^*t*^, *HIST2H2BE*^*r*, *t*^, *ITCH*^*n*, *V*^, *LAT*^*e*, *n*^, *MDM2*^*t*, *V*^, *MMP2*^*e*, *n*, *t*, *u*^, *NOTCH1*^*R*, *e*^, *NUP160*^*R*^, *PAIP1*^*R*^, *SDF4*^*R*, *e*,^ *UBC*^*r*, *t*, *V*, *v*, *g*^, *USP39*^*R*, *r*^, *ZAP70*^*e*, *n*, *g*^
*SMIM2-AS1*	small integral membrane protein 2 antisense RNA 1	13:44684685			0.00007	unknown
*RNASE12*	Ribonuclease A family member 12	14:21058240			0.00024	unknown
*RNASE8*	Ribonuclease A family member 8	14:21526052			0.00007	unknown
*YLPM1*	Tyrosine-Leucine-Proline motif-containing 1	14:75230069		0.00087	0.98413	*AKAP*^*p*^, *BMP1*^*R*, *t*^, *CDC5L*^*e*, *p*, *n*, *g*, *r*^, *CHD3*^*g*, *t*^, *DAB2*^*n*^, *DDX17*^*R*, *e*, *p*, *n*, *g*, *r*, *t*^, *DROSHA*^*g*^, *FU*^*r*, *t*^, *GRB2*^*n*, *p*, *t*^, *HNRNPA1*^*e*, *p*, *n*, *g*, *r*^, *ITSN2*^*R*^, *KHDRBS1*^*R*, *e*, *n*, *r*^, *KHSRP*^*e*, *p*, *n*, *g*, *v*^, *MAGEE1*^*t*^, *MATR3*^*R*, *e*, *p*, *n*, *g*, *r*, *t*, *v*^, *NCK2*^*R*^, *NONO*^*R*, *e*, *p*, *n*, *g*, *r*, *v*^, *PCGF1*^*R*^, *PPP1CA*^*t*^, *PPP1CB*^*e*, *t*^, *Ppp1cb*^*e*, *t*^, *PPP1CC*^*t*^, *PRMT1*^*g*^, *PRPF40A*^*e*, *p*, *n*, *g*^, *PRPF40A*^*e*, *p*, *n*, *g*^, *PSPC1*^*r*^, *RB1CC1*^*R*^, *SFPQ*^*e*, *p*, *n*, *g*, *r*^, *SMARCC1*^*t*^, *SMARCC2*^*t*^, *SRSF1*^*e*, *p*, *n*, *g*, *r*, *t*^, *TAF15*^*t*^
*C14orf80*	Chromosome 14 open reading frame 80	14:105956192		0.00018	0.65079	*ALOX5*^*e*^, *CDC16*^*R*^, *DDIT3*^*e*, *t*^, *JUNB*^*R*, *t*, *u*, *g*^, *KRAS*^*e*, *t*^, *SMC3*^*t*, *v*^, *TUBE1*^*t*^, *XPO1*^*R*, *g*, *r*, *t*, *v*, *u*^
*TMEM121*	Transmembrane protein 121	14:105992953			3.00E-06	unknown
*SRP14*	Signal recognition particle 14	15:40327891	Involved in arresting polypeptide formation in the ribosome complex and regulation of translation of cellular and viral RNAs including HI.[67–69]	0.00091	0.73016	g, t
*MIR6769A*	microRNA 6769a	16:4721319	The expression of many microRNAs differs by HIV status and disease characteristics and polymorphisms of microRNA genes is known to influence the outcomes of several viral infections.[55, 56]		0.00002	unknown
*MIR5010*	microRNA 5010	17:40666206	The expression of many microRNAs differs by HIV status and disease characteristics and polymorphisms of microRNA genes is known to influence the outcomes of several viral infections.[55, 56]		0.00003	unknown
*MIR6784*	microRNA 6784	17:43191735	The expression of many microRNAs differs by HIV status and disease characteristics and polymorphisms of microRNA genes is known to influence the outcomes of several viral infections.[55, 56]		0.00007	unknown
*ARMC7*	Armadillo repeat-containing 7	17:73106082	HIV *vpr* is known to bind to a related armadillo repeat, though the function of AMC7 is not known.[70]	0.00068	0.87302	*APP*^*e*, *t*, *p*^, *CKAP4*^*e*^, *CPSF6*^*R*, *r*, *g*, *p*^, *CPSF7*^*e*, *p*, *n*, *g*, *r*^, *EFHC2*^*R*^, *IKZF1*^*t*^, *IKZF3*^*V*, *p*^, *KCTD13*^*p*^, *PHB*^*e*, *p*, *n*, *g*, *t*, *v*^, *PHB2*^*e*, *p*, *n*, *g*^, *TRIM27*^*R*^, *TRIM42*^*R*^
*ZNF24*	Zinc finger protein 24	18:32912178	Member of a family of transcription factor that regulate cellular proliferation and differentiation angiogenesis and neural cell growth.[71, 72]	0.00083	0.02654	*CCDC8*^*R*, *g*^, *COPS6*^*v*^, *DDX6*^*e*, *g*, *p*^, *EEF1A1*^*R*, *e*, *p*, *n*, *g*, *r*, *t*^, *EEF1G*^*e*, *p*, *n*, *g*^, *ESR2*^*e*, *t*^, *FHL5*^*t*^, *HAP1*^*R*^, *HMGB1*^*R*^, *KAT5*^*t*, *g*^, *KBTBD7*^*R*^, *LRIF1*^*R*^, *MAPK6*^*e*^, *NFATC2*^*e*, *n*, *t*, *v*, *u*, *p*^, *PPP1CC*^*t*^, *PRKCQ*^*e*, *n*, *t*, *v*, *u*, *p*^, *PRPF31*^*r*^, *PSMA2*^*R*, *t*, *V*, *p*^, *PSMD1*^*e*, *t*, *V*, *p*^, *PSMD4*^*R*, *t*, *V*, *v*, *p*^, *SEC62*^*e*^, *SETDB1*^*R*, *t*^, *TNRC6A*^*n*^, *TP53*^*R*, *e*, *n*, *g*, *r*, *t*, *V*, *v*, *u*, *p*^, *TP53*^*R*, *e*, *n*, *g*, *r*, *t*, *V*, *v*, *u*, *p*^, *TRIM25*^*R*, *g*^, *TUBA3C*^*e*, *g*, *r*, *t*, *p*^, *UBE2I*^*e*, *g*, *p*^, *UNC119*^*n*^, *USP11*^*t*^, *YY1*^*t*, *p*^, *ZBTB16*^*V*^
*CCL25*	C-C motif chemokine ligand 25	19:8117646	Blood levels of gene product were related to HIV disease progression rate [73], homing of lymphocytes to mucosa[74] and inflammation in colitis[75]. In the SIV model, low CCL25 is associated with lymphoid apoptosis.[76]	0.00093	0.00113	*ACKR2*^e^, CCR9^n,v^, CCR10^g-p^
*GMIP*	GEM interacting protein	19:19740282	A member of the Ras family of GTPases, GIMP is thought to contribute to development of CD4 cells[77] as well as development of neural tissues and disease[78]	0.00062	0.02461	*ESR2*^e,t^, *GEM*^t^, *GCK*^g^, *RHOA*^e,g,n,t,V^, *XPO1*^g,r^
*ZNF493*	Zinc finger protein 493	19:21579921	zinc finger DNA binding factors contribute to lymphocyte development, maturation and homeostasis [79].	0.00082	0.85714	unknown
*KLC3*	Kinesin light chain 3	19:45843998	Member of a family of molecules that transport materials along microtubules, including immune response elements.	0.00087	0.80952	*ACBD5*^V^, *BAG6*^v^, *CCNB1*^e,t,v,V^, *CCNH*^t,V^, *CUL2*^v^, *CETN2*^g,V^, *DAPK2*^R^, *DYNC1I1*^g^, *GEM*^t^, *ITGB4*^t, g-p^, *KDM1A*^t^, *KIF5B*^g^, *LYST*^n^, *NFKB2*^e,t,V^, *NOLC1*^g^, *QARS*^g^, *RBBP6*^g^, *SF1*^g^, *SMARCB1*^n,g,t,g-p^, *YWHAE*^e,g-p,V^, *YWHAG*^g-p,t,V^, *YWHAH*^V^, *YWHAQ*^e,V^
*GCAT*	Glycine C-acetyltransferase AKA KBL	22:38203912	This nuclear gene encodes for a mitochondrial enzyme that converts threonine to glycine. Previous reports link NEMPs to AIDS progression, but not this gene.[80]	0.00058	1.00000	*FBXO6*^e^, *MCCC1*^g^, *MDM2*^t,V^
**Candidate Gene Design**						
*BCL2L11*	BCL2-like 11 or BCL-2 interacting mediator of cell death or *BIM*	2:111878491	Gene encodes for a protein that promotes apoptosis of T and B cells, and contributes to regulation of NK memory [81]. Polymorphisms are associated with several cancers, including lymphomas. [82, 83] HTLV factors interact with this gene [84].	0.15816	0.92063	t
*CCR5*	C-C motif chemokine receptor 5	3:46411633	A major HIV receptor, homozygote deletion mutants are resistant to infection and heterozygotes have slower disease progression. Genotype is associated with extent of CD4 recovery on cART.[23]		0.73016	e, g, n, t, v
*TNFSF10*	Tumor necrosis factor superfamily member 10 AKA TRAIL/CD253 (tumor necrosis factor-related apoptosis-inducing ligand)	3:172223298	Gene product is a B cell surface molecule related to lymphocyte apoptosis and survival in conjunction with other factors. Polymorphisms previously reported to influence CD4 recovery during ART.[21]	0.28529	0.55319	e, t, V, v
*IL15*	Interleukin 15	4: 142557749	Gene product is a cytokine that regulates T cell function, polymorphisms are linked to a wide range of conditions, including the outcomes of several infections and occurrence of cancer.	0.57523	0.31200	e, g, n, p, t
*IL7R*	Interleukin 7 receptor	5: 35856977	IL-7 is a major homeostatic regulator of CD4 lymphocytes. Polymorphisms of the IL7 receptor gene can result in SCID and rapid progression of HIV disease [85] and poor CD4 recovery on cART.[22, 25]		0.08902	t, V
*TNF*	tumor necrosis factor	6: 31543344	Gene encodes for a major inflammatory mediator. Polymorphisms are associated with multiple inflammatory diseases, hypersensitivities and infections.	0.7263	0.08712	e, g, n, t, u, v
*IL7*	Interleukin 7	8: 79645007	Gene encodes for a major homeostatic regulator of T and B cells.	0.36891	0.66667	e, g, p, t
*IL15RA*	Interleukin 15 receptor subunit alpha	10: 5994334	Gene encodes a high affinity IL15 receptor. Polymorphisms previously reported to influence CD4 recovery during ART.[21]		0.84127	e

^1^ Adjusted for genomic estimates of race and ethnicity and age (years). HIV protein interactions with the gene of interests was categorized as unknown, direct (i.e., an HIV protein(s) interacts directly with the protein product encoded by the gene of interest), or indirect (i.e., the protein encoded by the gene of interest interacts with another host protein that in turns is known to interact with an HIV viral protein(s)). For direct HIV protein interactions, the HIV protein is denoted with a single letter designation (i.e., e = Env; g = Gag protein; n = Nef; p = Pol; t = Tat protein; r = reverse transcriptase; u = Vpu; V = Vif; v = Vpr protein); R = knockdown of the host protein results in impaired HIV-1 replication. For indirect HIV protein interactions, the host protein that interacts with the protein encoded for the protein of interest is listed by its HUGO gene identifier and the HIV protein denoted with a single letter designation (e.g., v = Vpr protein). Abbreviations: ART = HIV antiretroviral therapy; cART = combination ART; CNS = central nervous system; CMC = Combined Multivariate and Collapsing regression analysis; HTLV = human T-cell lymphotropic virus; KBAC = Kernel-Based Adaptive Collapsing regression analysis; NEMP = Nuclear Envelope integral Membrane Protein; n/a = not available; NK = natural killer cell memory; Position = chromosome and position; SCID = severe combined immunodeficiency.

## Discussion

Several predictors of CD4 cell response group among suppressed (by clinical assay) cART recipients were identified in this study. Of the non-genetic factors, only age was statistically significantly associated with CD4 cell response group in multivariable analysis. Greater age was associated with lower likelihood of a good CD4 recovery response, a finding that has been previously reported.[86, 87] This is in keeping with the loss of immune function that is known to occur with aging

Some studies have reported that females have higher circulating numbers of CD4 lymphocytes than males[88, 89], but substantial sex differences in CD4 cells have not been consistently observed.[90] Some reports note that sex differences in CD4 cell counts is driven by higher counts among women less than 50 years of age.[89] We found that a biomarker of gonadal aging (ovarian follicular function) was associated with recovery group, but the association was not statistically significant when adjusted for chronological age.

Many of the genes identified in this study appear to participate in processes related to lymphocyte homeostasis and thymus activity. Some genes that are known to interact with HIV also were associated with CD4 lymphocyte response group.

The two approaches we employed to evaluate genetic burden are related, but provide distinct information. While each analysis estimates genetic burden, CMC evaluates both rare and common polymorphisms and identified 23 genes that suggests that the basis of these associations is due primarily to common and not rare variant burden, while KBAC evaluates rare polymorphisms and identified 17 genes that suggests that the basis of these associations is due primarily to rare variant burden. Only one gene was identified by both CMC and KBAC (i.e., *CCL25*), indicating that both rare and common variants of this gene may influence CD4 cell recovery pattern.

Further investigation of the 41 positional candidate genes revealed that 9 encoded for RNA products, while the remainder encoded for proteins. Twenty-five of the 32 protein-coding genes encoded for proteins that interacted with HIV proteins (**Table 2**, last column). The 9 RNA-encoding genes have not been evaluated for their potential HIV protein interactions. However, the expression of microRNAs often differs by HIV status and disease characteristics.

In a post-hoc analysis, we identified publically available gene expression data collected in a sample of South Asian men (n = 10) and women (n = 2) who exhibited expected (n = 5) and poor (n = 7) CD4 T-cell recovery after cART initiation (Gene Expression Omnibus [GEO] expression set GSE77939) which we interrogated to determine if a subset of the 41 genes that were enriched for sequence variations associated with poor CD4 cell recovery also exhibited differences in gene expression. The definitions of expected and poor CD4 cell recovery were loosely similar to ours, with HIV-positive individuals required to take ART for at least one year who did (expected responders) and did not (poor responders) experience a CD4 lymphocyte count gain of more than 150 cells/μL in one year and a viral load <400 copies/mL). Additionally, non-responders consistently had CD4 cell counts of less than 250 cells/uL, while expected responders showed a CD4 count that exceeded 250 cells/μL (personal communication, S. Aurora). Gene expression measures for 7 (i.e., *CTAGE7P*, *MIR3662*, *MIR5010*, *MIR6769A*, *MIR6784*, *RP11-383C5*.*3*) of the 41 candidate genes identified in our sample were not available. Of the 34 genes that we identified by whole exome sequencing for which gene expression data was also available, seven were differentially expressed between CD4 responder groups (i.e., *ALG2*, *GIMP*, *GRHL2*, *LCE1D*, *SLC12A2*, *TMEM121*, *ZNF493*; all p<0.05) based on GEO2R analysis. These gene expression findings provide complementary support that the identified candidate genes that differed in terms of enrichment of sequence variations by CD4 cell recovery group also show differences in gene expression. Whereas the number of genes that were differentially expressed exceed that expected by chance (i.e., 7 genes were differentially expressed where approximately 2 would be expected by chance alone, p<0.00022), it is also possible that a subset of the other candidate genes may also be differentially expressed and associated with CD4 cell response to ART but in other tissues (e.g., thymus) or at different times (e.g., viral load set-point). Future studies are warranted that examine gene expression and sequence variation (e.g., expression-quantitative trait locus) analyses in relation to CD4 lymphocyte response to treatment.

Further bioinformatic analyses to evaluate for a biological pattern(s) across the genes were identified using KEGG gene set enrichment analyses. One pathway, hsa00260 (glycine, serine, and threonine metabolism) featured two genes associated with CD4 recovery: *CDCH* and *GCAT*. Enrichment of genes in the hsa00260 pathway has been highlighted in several studies of HIV infection.[91, 92] Because we identified HIV-related genes that were associated with CD4 cell recovery pattern, it is possible that ongoing HIV replication at very low levels might have been a contributor to CD4 cellular expansion. Thus, when specimens were available, we conducted ultrasensitive HIV RNA assays to detect HIV RNA quantities below the threshold of clinical assays in use at the time of this study. These ultra-sensitive assays utilized Hologic (San Diego, CA) using the Hologic Aptima® HIV-1 Quant DX Assay on the Panther System with up to 9 replicates of each sample and can detect HIV RNA levels as low as 1 copy/ml. Thirty-eight of 43 good CD4 cell responders with available samples for testing had ≥1 replicate test that had detectable HIV RNA. All 10 of the available poor CD4 cell responder samples had detectable HIV RNA in ≥1 replicate. This finding indicates that persistent viral replication exists, even among individuals thought to have optimal viral suppression. However, despite the frequency of ongoing low-level replication, most cART recipients experience rapid expansion of CD4 lymphocyte populations. It is possible that individuals vary in terms of how on-going low-level viral replication impacts CD4 recovery. The fact that some of the variations in genes we found to be predictive of CD4 response group had known relationships with HIV also supports a continuing role of HIV replication in the setting of host differences in determining immunologic recovery.

## Conclusions

This study confirms the finding that increasing age is an independent and strong predictor of poor CD4 recovery during cART. We found that age, in a group of mid-life women, was a better predictor of CD4 recovery than AMH, a biomarker of gonadal aging. This result suggests that loss of ovarian estrogen is not a leading determinant of CD4 recovery during cART. A major contribution of this study, which broadly searched for relationships between host genetics and CD4 cell recovery, is the identification of new genes that may influence immune reconstitution in treated HIV infection. These findings may lead to novel hypotheses and identification of new host factors which contribute to HIV pathogenesis and recovery.

## Supporting information

S1 Fig Flow diagram of variant calling file generation. Object shapes indicate programs (ovals) or data files (rectangles). Arrows connect program inputs and outputs. Rectangle color indicates the type of data: study data (green), reference data (blue), and intermediate processed data (maroon). Abbreviations: 1000G = 1000 Genomes; dbSNP = Nartional Center for Biotechnolgy Institute SNP database; GATK = Genome Analysis Tookit from The Broad Institute; Hapmap = The haplotype map database (www.hapmap.org); hg19 = Human Genome UCSC assembly 19; SNP = single nucleotide polymorphism; VCF = Variant Calling Format.
(TIFF)

S1 Table Detailed estimates of gene-wise associations using CMC and KBAC. The study design is listed in column A and is entitled “StudyDesign” which has one of two values: “Novel Loci” identified by exome sequencing and “Candidate Gene” because the given gene was specified *a priori*. The gene abbreviation is specified in column B. The chromosome location of the gene is listed in column C. The gene identifier is listed in column D. The gene name is defined in column E. The nucleotide start position for the gene is listed in column F. The nucleotide end position for the gene is listed in column G. The gene transcript identifier is listed in column H. The strand (i.e., “+”, “-“) encoding the gene is listed in column I. The total number of markers (SNPs) included in the CMC-based associated test is listed in column J. The number of markers included in each of the five possible bins for the CMC-based test of association are listed in columns K through O (i.e., CMC Bin_0_ Count, CMC Bin_1_ Count, CMC Bin_2_ Count, CMC Bin_3_ Count, CMC Bin_4_ Count). The unadjusted p-value for the omnibus CMC-based test of association for each gene is provided in column P. The unadjusted estimate of association (beta and associated standard error [SE]) for the omnibus CMC-based test of association is listed in column Q. The unadjusted estimate of association (beta and associated standard error [SE]) for the first bin (“0”) is listed in column R. The unadjusted estimate of association (beta and associated standard error [SE]) for the second bin (“1”) is listed in column S. The unadjusted estimate of association (beta and associated standard error [SE]) for the third bin (“2”) is listed in column T. The unadjusted estimate of association (beta and associated standard error [SE]) for the fourth bin (“3”) is listed in column U. The unadjusted estimate of association (beta and associated standard error [SE]) for the fifth bin (“4”) is listed in column V. The Chi-squared value statistic for the likelihood ratio test of the adjusted estimate of association as compared to the unadjusted estimate of association, when adjusting for genomic estimates of race and ethnicity (i.e., principal component 1 [PC1], principal component 2 [PC2]), is listed in column W. The adjusted estimate of association (beta and associated standard error [SE]), when adjusting for genomic estimates of race and ethnicity (i.e., principal component 1 [PC1], principal component 2 [PC2]) and age, for the omnibus CMC-based test of association is listed in column X. The adjusted estimate of association (beta and associated standard error [SE]) is listed in column Y. The adjusted estimate of association (beta and associated standard error [SE]) for PC1 is listed in column Z. The adjusted estimate of association (beta and associated standard error [SE]) for PC2 is listed in column AA. The adjusted estimate of association (beta and associated standard error [SE]) for age is listed in column AB. The adjusted estimate of association (beta and associated standard error [SE]), for the first bin (“0”) is listed in column AC. The adjusted estimate of association (beta and associated standard error [SE]), for the second bin (“1”) is listed in column AD. The adjusted estimate of association (beta and associated standard error [SE]), for the third bin (“2”) is listed in column AE. The adjusted estimate of association (beta and associated standard error [SE]), for the fourth bin (“3”) is listed in column AF. The adjusted estimate of association (beta and associated standard error [SE]), for the fifth bin (“4”) is listed in column AG. The Chi-squared value statistic for the likelihood ratio test of the adjusted estimate of association as compared to the unadjusted estimate of association, when adjusting for genomic estimates of race and ethnicity (i.e., principal component 1 [PC1], principal component 2 [PC2]), and age, and non-adherence, is listed in column AH. The adjusted estimate of association (beta and associated standard error [SE]), when adjusting for genomic estimates of race and ethnicity (i.e., principal component 1 [PC1], principal component 2 [PC2]) and age, for the omnibus CMC-based test of association is listed in column AI. The adjusted estimate of association (beta and associated standard error [SE]) is listed in column AJ. The adjusted estimate of association (beta and associated standard error [SE]) for PC1 is listed in column AK. The adjusted estimate of association (beta and associated standard error [SE]) for PC2 is listed in column AL. The adjusted estimate of association (beta and associated standard error [SE]) for age is listed in column AM. The adjusted estimate of association (beta and associated standard error [SE]) for non-adherence is listed in column AN. The adjusted estimate of association (beta and associated standard error [SE]) for adherence is listed in column AO. The adjusted estimate of association (beta and associated standard error [SE]), for the first bin (“0”) is listed in column AP. The adjusted estimate of association (beta and associated standard error [SE]), for the second bin (“1”) is listed in column AQ. The adjusted estimate of association (beta and associated standard error [SE]), for the third bin (“2”) is listed in column AR. The adjusted estimate of association (beta and associated standard error [SE]), for the fourth bin (“3”) is listed in column AS. The adjusted estimate of association (beta and associated standard error [SE]), for the fifth bin (“4”) is listed in column AT. The total number of markers (SNPs) included in the KBAC-based associated test is listed in column AU. The number of multi-marker genotypes estimated for the KBAC test is listed in column AV. The unadjusted p-value for the KBAC-based test of association for each gene is provided in column AW. The KBAC test statistics (i.e., the KBAC score) for the unadjusted estimate of association is listed in column AX. The adjusted (i.e., genomic estimates of race and ethnicity [PC1, PC2], age) p-value for the KBAC-based test of association for each gene is provided in column AY. The KBAC test statistics (i.e., the KBAC score) for the PC1-, PC2-, and age-adjusted estimate of association is listed in column AZ. The adjusted (i.e., genomic estimates of race and ethnicity [PC1, PC2], age, self-reported non-adherence) p-value for the KBAC-based test of association for each gene is provided in column BA. The KBAC test statistics (i.e., the KBAC score) for the PC1-, PC2-, age-, non-adherence-adjusted estimate of association is listed in column BB.
(XLSX)

S2 Table Single nucleotide polymorphisms incorporated into each gene burden analysis. The study design is listed in column A and is entitled “StudyDesign” which has one of two values: “Novel Loci” identified by exome sequencing and “Candidate Gene” because the given gene was specified *a priori*. The chromosome location of the gene is listed in column B. The gene abbreviation is specified in column C. The gene identifier is listed in column D. The nucleotide position is listed in column D. The RefSeq identifier (rsID) is provided, if available, in column E. The reference allele is provided in column F. The excess heterozygosity ratio statistic, *R*, is listed in column G. The p-value for Fisher’s Exact test for the association of a given SNP with the CD4 Recovery Group outcome is listed in column H. The odds ratio (OR) and the 95% confidence interval (95% CI) of the association of a given SNP with the CD4 Recovery Group outcome is listed in column I. The sample size is listed in column J. The SNP call rate is listed in column K. The reference minor allele frequency (MAF) is provided in column L. The p-value for the Fisher’s Exact Test for the deviation of a given SNP from the Hardy-Weinberg expectation is listed in column M.
(XLSX)

S1 Appendix SAS programming. (PDF)

S2 Appendix Detailed specification of the phenotyping algorithm. (PDF)
