# Supplementary figures and images for: Genetic and clinical predictors of CD4 lymphocyte recovery during suppressive antiretroviral therapy: Whole exome sequencing and antiretroviral therapy response phenotypes
Source: PLoS One. 2019 Aug 15;14(8):e0219201. doi: 10.1371/journal.pone.0219201 (PMC6695188; doi:10.1371/journal.pone.0219201)

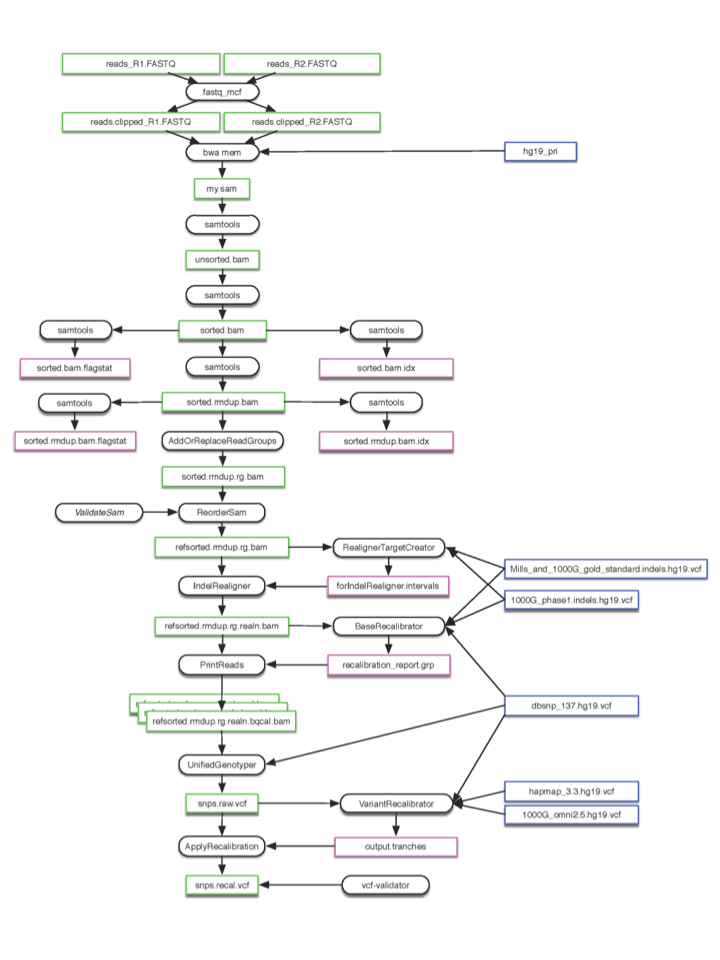

Supplement: S1 Fig — Object shapes indicate programs (ovals) or data files (rectangles). Arrows connect program inputs and outputs. Rectangle color indicates the type of data: study data (green), reference data (blue), and intermediate processed data (maroon). Abbreviations: 1000G = 1000 Genomes; dbSNP = Nartional Center for Biotechnolgy Institute SNP database; GATK = Genome Analysis Tookit from The Broad Institute; Hapmap = The haplotype map database (www.hapmap.org); hg19 = Human Genome UCSC assembly 19; SNP = single nucleotide polymorphism; VCF = Variant Calling Format. (TIFF) [file pone.0219201.s001.tiff]
