# Supplementary material for: Genetic and clinical predictors of CD4 lymphocyte recovery during suppressive antiretroviral therapy: Whole exome sequencing and antiretroviral therapy response phenotypes
Source: PLoS One. 2019 Aug 15;14(8):e0219201. doi: 10.1371/journal.pone.0219201 (PMC6695188; doi:10.1371/journal.pone.0219201)
Supplement: S1 Appendix — (PDF) [file pone.0219201.s004.pdf]

SF WIHS Phenotyping Project – Programming Specifications

The goal of this project is to characterize the history of women with HIV in WIHS. The key variables refer to HIV treatment status, CD4 counts, and HIV viral load. Before characterizing the history we must deal with missing or noisy data, which are widespread problems.

Unless otherwise noted, apply the steps below in the order given.

Note that this document uses the term "HAART" to denote combination antiretroviral therapy (cART).

Preliminaries

The original data are irregular, with missing visits and missing observations within visits. They also have data we want to ignore. The goal of preliminary processing is to create a regularized data set for all later operations. These data form a complete grid of visits by variable, though some of the cells have missing data (as opposed to the earlier form, in which the cells were completely absent).

All rules in later sections should be interpreted in the context of these regularized data. For example, if a woman missed visit 6, her "next visit" after visit 5 still means visit 6 in the regularized data.

1. Fill in all visits and observations absent from the original data with observations whose value is missing ('.' in SAS).
2. Analysis will focus on a particular range of visits for each woman. However, the regularized data set includes "buffer visits" before and after the analytic range to avoid having to treat the regions near the endpoints with special rules and code.
3. The **analytic first visit** is
    a. For sero-converters, the first visit the woman was known HIV+.
    b. For others, the first regular visit. This excludes screening visits.
4. The **analytic last visit** is the last observed visit.
5. The regularized data include a buffer from 4 visits before the analytic first visit through 4 visits after analytic last visit. E.g., if a woman has an analytic range of visits 1 through 36, the extended range will be from -3 through 40. All data are missing (SAS '.') for the buffer visits.

Interpolation and Smoothing

Missing results can be filled in under some circumstances, and not in others. CD4 readings are erratic and so we smooth them. Only update visits between the analytic first visit as defined in rule 3 and the last observed visit (rule 4).

6. Create new variables phenoThrpy, phenoVL, phenoUndetVL, and phenoCD4 to start out as equal to the corresponding observed values of THRPYV, HIV viral load, an indicator for viral load undetectable (regardless of what the detection threshold was), and CD4 count.
7. Recode phenoCD4 to have a maximum value of 1,000, since variation among high values is of no interest to us.
8. If one or two visits with THRPYV missing are preceded and followed by visits with THRPYV known, and those visits have the same value for THRPYV, then impute phenoThrpy for the missed visits as equal to that value. Otherwise, leave phenoThrpy missing.
9. All subsequent interpolation and smoothing rules only apply if the relevant window has visits that are all of the same, known, phenoThrpy. **Do not interpolate across different or unknown treatments.**
10. Viral Load (phenoVL, phenoUndetVL).
    a. If one or two visits with missing VL are preceded and followed by visits where viral load is below the detection limit, then impute that visit as having phenoUndetVL=1, phenoVL= detection limit (lower of the two).
    b. Refer to the visits with non-missing viral information as **bracketing visits**.
    c. If one of the bracketing visits has undetectable VL and one has VL detectable but <1000, then impute phenoUndetVL=1, phenoVL= detection limit.
    d. If one of the bracketing visits has undetectable VL and one is detectable and >=1000, then leave phenoUndetVL= missing and phenoVL= missing.
    e. For imputation of viral load between bracketing visits both of which have detectable viral loads, set phenoUndetVL=0 and use

    $$VL = VL0\left(\frac{VL1}{VL0}\right)^{\frac{v-v0}{v1-v0}}$$

    for phenoVL, where VL = viral load to impute (phenoVL), VL0=viral load of the earlier visit, VL1=viral load of the later visit, v=visit number of the imputed visit, v0=visit number of the earlier visit, v1=visit number of the later visit. This is the value assuming geometric growth.
    f. If there are 3 or more consecutive visits with missing viral load, then leave phenoUndetVL and phenoVL as missing for all those visits.
11. CD4 (phenoCD4).
    For each "current" visit, define a "smoothing window". Recall that phenoThrpy must have a known, constant value across the entire window (rule 9).
    a. Even if the previous visit is not in the smoothing window, the previous visit must have the same phenoThrpy as the window in order to smooth. This requirement does not apply to the first analytic visit, which may be smoothed.
    b. If the current CD4 is not missing, use the following table to determine which visits to use together with the current visit in the smoothing. An X indicates a non-missing value; use the first row that matches and satisfies constant, known phenoThrpy. The columns are the indicated number of visits before or after the visit to be smoothed.

	-4	-3	-2	-1	+1	+2	+3	+4
i				X	X			
ii			X		X			
iii				X		X		

iv			X			X		
v			X	X				
vi		X		X				
vii	X			X				
viii		X	X					
ix	X		X					
x					X	X		
xi					X		X	
xii					X			X
xiii						X	X	
xiv						X		X

c. If the current CD4 is missing, using the following table to determine which visits to use for interpolation (see above for interpretation).

	-4	-3	-2	-1	+1	+2	+3	+4
i			X	X	X			
ii				X	X	X		
iii		X		X	X			
iv				X	X		X	
v	X			X	X			
vi				X	X			X
vii			X	X		X		
viii		X		X		X		
ix	X			X		X		
x		X	X		X			
xi	X		X		X			
xii		X	X			X		
xiii	X		X			X		
xiv			X		X	X		
xv			X		X		X	
xvi			X		X			X
xvii				X		X	X	
xviii				X		X		X
xix			X			X	X	
xx			X			X		X
xxi				X	X			
xxii				X		X		
xxiii			X		X			
xxiv			X			X		

d. If one of the smoothing windows above is applicable, define sqrtCD4 = sqrt(CD4). Fit a regression line to the 2 or 3 observations in the window by modeling sqrtCD4 in terms of visitnum, define fittedRootCD4 as the fitted value at the focal visit, and set phenoCD4=fittedRootCD4*fittedRootCD4.

e. If none of windows are possible, then leave phenoCD4 = observed CD4 (no smoothing). Note that if observed CD4 is missing in this case, then phenoCD4 will also be missing (no filling in will be possible).

History

Define the following variables describing history up through the focal visit:

12. phenoHAART gives the number of consecutive visits on HAART
    a. phenoHAART=0 if .<phenoThrpy<3
    b. phenoHAART=1 if phenoThrpy=3 and the previous visit has .<phenoThrpy<3
    c. phenoHAART= previous visit's phenoHAART + 1 if phenoThrpy=3 and previous visit has phenoHAART>0
    d. phenoHAART=missing if phenoThrpy-missing
    e. phenoHAART=missing if phenoThrpy=3 and previous visit has phenoHAART=missing. This rule does not apply if the previous visit precedes the first analytic visit.
    f. phenoHAART=missing if phenoThrpy=3 and this is the first analytic visit, unless the exceptions noted next (12.g) apply.
    g. phenoHAART=1 if first analytic visit and phenoThrpy=3 and either
        i. woman is a first-wave recruit
        ii. woman reports no previous HAART therapy and the visit of that report is no more than 1 visit before the focal visit.
        iii. woman is a seroconverter and the visit on which she was HIV- was the previous visit.
    h. Note that phenoThrpy=4 is documented in the codebook, but not used in the data.
13. phenoHAART2 gives the minimum number of consecutive visits on HAART. In some cases we know this even if we do not know the actual number of visits on HAART.
    a. phenoHAART2=0 if .<phenoThrpy<3
    b. phenoHAART2=1 if phenoThrpy=3 and the previous visit has .<phenoThrpy<3 or missing.
    c. phenoHAART2=2 if phenoThrpy=3 and the previous visit has phenoHAART2=1
    d. phenoHAART2= previous visit's phenoHAART2 + 1 if phenoThrpy=3 and previous visit has phenoHAART2>0
    e. phenoHAART2=missing if phenoThrpy is missing
    f. phenoHAART2=1 if first analytic visit and phenoThrpy=3.
14. phenoCD4nadir is the minimum phenoCD4 over all the study visits up to and including the focal visit. Exclude missing values from this calculation, so that phenoCD4nadir will only be missing if all phenoCD4 values up to the focal visit are missing.
15. phenoOffHAART gives the number of consecutive visits not on HAART. It is missing if not known exactly.
    a. phenoOffHAART = 0 if phenoThrpy=3
    b. phenoOffHAART = missing if phenoThrpy is missing.and visit > 4.
    c. phenoOffHAART = missing on the initial visit unless a. applies
    d. phenoOffHAART = previous phenoOffHAART + 1 (and so is missing if the previous phenoOffHAART is missing) if after the initial visit and either
        i. . < phenoThrpy<3 or
        ii. phenoThrpy is missing and Visit ≤ 4
16. "baseline CD4" is the average CD4 level while on HAART (phenoThrpy=3) immediately before visits off HAART.
    a. The average of the 2 previous phenoCD4 if both visits are on HAART with non-missing phenoCD4.
    b. The available phenoCD4 if one of the 2 visits on HAART has a non-missing value.
    c. The phenoCD4 if only a single visit off HAART precedes the visits off HAART.

Phenotypes

We classify visits into phenotypes based on whether or not someone is on HAART, how long they have been on or off HAART, viral load and CD4. The phenotype has one code for each aspect, in order. A full table of phenotype names and symbols appears at the end of this document, but the following table provides an overview:

ASPECT	CODE	MEANING
Treatment	H	On HAART
	S	Short-term off HAART
	L	Long-term off HAART
	Various	Unclassifiable or HIV-
Viral Load	1	Undetectable, or mostly so
	2	Low viremic
	3	Viremic
	0	Too early to tell (first visit)
CD4	a	Good
	b	Bad
	x	Can't tell

For example, H1a is the phenotype for visits of woman who are on HAART with undetectable viral load and good CD4 counts. Thresholds vary based on treatment history, as detailed below.

17. Group L (Long-term off HAART) will include HIV+ women's visits that have phenoThrpy=0, 1, or 2, i.e. visits not on HAART,, for all visits in the definition window. Ordinarily the window is the current visit and the 6 previous ones. However, if a woman with no pre-enrollment HAART enters the study the window is shortened to all previous study visits, provided there are at least 2 prior visits in the study.

    a. L1 are viral elite controllers
       All variants of this phenotype must have phenoUndetVL=1 for all, or all but one, visits in window. The exceptional visit may have missing phenoVL or have phenoUndetVL=0 and .<phenoVL<=1000. The CD4 requirements for specific phenotypes follow.
        i. L1a: phenoCD4nadir>=500.
        ii. L1b: .<phenoCD4nadir<500.
        iii. L1x: phenoCD4nadir=missing.
    b. L2 are low viremic controllers
       Every visit in window, or all but one visit in window, has either phenoUndetVL=1 or .<phenoVL<=2000. Note the one visit for which the condition is false may have a missing phenoVL. Exclude those satisfying the VL condition for L1. CD4 requirements for specific phenotypes follow.
        i. L2a: phenoCD4nadir>=500.
        ii. L2b:.<phenoCD4nadir<500.
        iii. L2x: phenoCD4nadir=missing.
    c. L3
       At least two visits in window have phenoUndetVL=0 & phenoVL>2000.
        i. L3a:. phenoCD4nadir>=500.
        ii. L3b: .<phenoCD4nadir<500.
        iii. L3x: phenoCD4nadir=missing.

18. Group S (Short-term off-HAART) will include visits when women have been off HAART for shorter than the L group, but more than one visit. Visits for which there is no prior visit on HAART in the study are ineligible for this classification; in particular, report of pre-enrollment HAART is insufficient to establish eligibility. See sections 15 and 16 for definitions of baseline CD4 and phenoOffHAART
    a. S1
       All variants of this phenotype must have phenoUndetVL=1 for all, or all but one, visits in window. The exceptional visit may have missing phenoVL or have phenoUndetVL=1 and .<phenoVL<=1000.

        i. S1a: phenoCD4>=(baseline CD4)*(1-10/100)**(phenoOffHAART). [This defines <10% deterioration per visit in CD4 as "good".]
        ii. S1b: .<phenoCD4<(baseline CD4)*(1-10/100)**(phenoOffHAART).
        iii. S1x: phenoCD4 missing or all phenoCD4 for computing baseline CD4 are missing.
    b. S2
       Every visit in window, or all but one visit in window, has either phenoUndetVL=1 or .<phenoVL<=2000. Exclude those satisfying the VL requirements for S1. Define "baseline CD4" as in S1.
        i. S2a: phenoCD4>=(baseline CD4)*(1-10/100)**(phenoOffHAART).
        ii. S2b: .<phenoCD4<(baseline CD4)*(1-10/100)**(phenoOffHAART).
        iii. S2x: phenoCD4 missing or all phenoCD4 for computing baseline CD4 are missing.
    c. S3
       >1 visit in window has phenoUndetVL=0 & VL>2000.
        i. S3a: phenoCD4>=(baseline CD4)*(1-10/100)**(phenoOffHAART).
        ii. S3b: .<phenoCD4<(baseline CD4)*(1-10/100)**(phenoOffHAART).
        iii. S3x: phenoCD4 missing or all phenoCD4 for computing baseline CD4 are missing.
19. Group H1 is for visits from HIV+ women on HAART, phenoThrpy=3, whose viral load is undetectable, phenoUndetVL=1.
    a. H1a are visits where women have good CD4 recovery/maintenance on HAART.
       When calculating mimima with missing phenoCD4nadir, ignore the nadir term, i.e., use 350 as the cutoff in this subsection only.
       1) PhenoHAART=1 or 2 and phenoCD4>= min(350, phenoCD4nadir+50)>.; or
       2) PhenoHAART=3 and phenoCD4>= min(350, phenoCD4nadir+100)>.; or
       3) PhenoHAART=4 and phenoCD4>= min(350, phenoCD4nadir+150)>.; or
       4) PhenoHAART>=5 and phenoCD4>= min(350, phenoCD4nadir+200)>. ; or
       5) PhenoHAART=. & phenoCD4>=min(350, phenoCD4nadir+200).
    b. H1b are visits with poor CD4 recovery/maintenance.
       This classification requires non-missing phenoCD4nadir.
       For this subsection only "HAARTvisits" = phenoHAART if that is defined, otherwise phenoHAART2. A visit is H1b if:
       1) HAARTvisits=2 and .<phenoCD4< min(350, phenoCD4nadir+50); or
       2) HAARTvisits=3 and .<phenoCD4< min(350, phenoCD4nadir+100); or
       3) HAARTvisits=4 and .<phenoCD4< min(350, phenoCD4nadir+150); or
       4) HAARTvisits>=5 and .<phenoCD4< min(350, phenoCD4nadir+200).
    c. H1x is for visits that do not clearly exhibit good or bad CD4 recovery. These are visits with non-missing phenoHAART or phenoHAART2, not classified by 19.a or 19.b
20. Group D1x is for visits on HAART, undetectable viral load, and with unknown phenoHAART, unless assigned above.
21. Group H0/H3 is for visits from HIV+ women with viral load above detection, phenoUndetVL=0, on HAART, phenoThrpy=3.
    a. H0x: first visit on HAART.

b. H3a: visits meeting the same criteria as for H1a above, except that phenoUndetVL=0.
c. H3b: visits meeting the same criteria as for H1b above, except that phenoUndetVL=0.
d. H3x: visits 1) with phenoHAART>1 and phenoUndetVL=0 and phenoCD4 missing.

22. Group D3x is for visits with all of phenoThrpy=3, phenoHAART=missing, phenoUndetVL=0 and phenoCD4< min(350, phenoCD4nadir+200) or phenoCD4=.

23. Group N is for visits from HIV uninfected women.

24. Group U is for all visits not classified above. This category includes, but is not limited to, those on mono/combo non-HAART therapy and blocks of 3 or more missed visits.

Post-Processing

25. Delete all visits before the first or after the last analytic visit.

Summary table of phenotypes

Treatment	Viral Load	CD4	Phenotype	Symbol in Graphs
Off tx*, all/7+ visits	Good	Good	L1a	**L**
Off tx, all/7+ visits	Good	Bad	L1b	**l**
Off tx, all/7+ visits	Good	Unknown	L1x	***L***
Off tx, all/7+ visits	Medium	Good	L2a	**L**
Off tx, all/7+ visits	Medium	Bad	L2b	**l**
Off tx, all/7+ visits	Medium	Unknown	L2x	***L***
Off tx, all/7+ visits	Bad	Good	L3a	**L**
Off tx, all/7+ visits	Bad	Bad	L3b	**l**
Off tx, all/7+ visits	Bad	Unknown	L3x	***L***
Off tx, 1-6 visits	Good	Good	S1a	**S**
Off tx, 1-6 visits	Good	Bad	S1b	**s**
Off tx, 1-6 visits	Good	Unknown	S1x	***S***
Off tx, 2-6 visits	Medium	Good	S2a	**S**
Off tx, 2-6 visits	Medium	Bad	S2b	**s**
Off tx, 2-6 visits	Medium	Unknown	S2x	***S***
Off tx, 2-6 visits	Bad	Good	S3a	**S**
Off tx, 2-6 visits	Bad	Bad	S3b	**s**
Off tx, 2-6 visits	Bad	Unknown	S3x	***S***
First HAART	Good	Good	H1a	**H**
First HAART	Good	other	H1x	***H***
First HAART	Bad	Any	H0x	***H***
>1st HAART	Good	Good	H1a	**H**
>1st HAART	Good	Bad	H1b	**h**
>1st HAART	Good	Unknown	H1x	***H***
>1st HAART	Bad	Good	H3a	**H**
>1st HAART	Bad	Bad	H3b	**h**
>1st HAART	Bad	Unknown	H3x	***H***
Unk HAART	Good	Good	H1a	**H**
Unk HAART	Good	Bad	H1b	**h**
Unk HAART	Good	Unknown	D1x	***D***
Unk HAART	Bad	Good	H3a	**H**
Unk HAART	Bad	Bad	H3b	**h**
Unk HAART	Bad	Unknown	D3x	***D***
Other, in study	Any	Any	U	**U**
In study	Unknown	Any	U	**U**
After last visit				blank
HIV negative			N	No plot

*Off tx means Off HAART

Key:
First position, Capital letter
L – Long-term off HAART
S – Short-term off HAART
H – on HAART
D – Unk HAART visit
1 – good VL 2 – low viremic 3 – Bad VL 0 – Can't tell yet (1st HAART visit)
a – good CD4 b – Bad CD4 x – Can't tell
N – HIV negative
U – unclassified (includes missed visits, mono/combo therapy)
