# Supplementary material for: Genetic and clinical predictors of CD4 lymphocyte recovery during suppressive antiretroviral therapy: Whole exome sequencing and antiretroviral therapy response phenotypes
Source: PLoS One. 2019 Aug 15;14(8):e0219201. doi: 10.1371/journal.pone.0219201 (PMC6695188; doi:10.1371/journal.pone.0219201)
Supplement: S2 Appendix — (PDF) [file pone.0219201.s005.pdf]

Material includes programming for phenotypes not reported in this paper

```
/*  Interpolate and build phenotypes for HIV treatment and response

      Authors: Peter Bacchetti, Chengshi Jin, Ross Boylan
      Finalized: April 10, 2016

      NOTE: Requires using the MS-Access and MS-Excel engines.

      References in comments like "Spec 4" refer to item 4 in the Appendix A.

      INPUTS
      wdbV40.mdb  WIHS Access databse

      benchmark.csv     test cases and expected results
      formats.sas       standard formats

      OUTPUTS
      p.pheno_20160410 interpolated, phenotyped data
            the visit variable here has been modified to be mostly calendar-based.

      p.visits    info for corrected visits
            wihsid
            visit as in original data
            visit2  corrected, calendar-based visit (missing for all but last in interval)
            visit_interval corrected visit number based on interval, never missing.

      p.step5    (for testing)

*/
%let test=0; * set to 1 to operate in self-test mode;
%let LASTVISIT=41;
options mprint; * since most of the code is inside macros, this makes it easier to see what is happening in SAS log
files;

/*** Generic options ***/
options nodate nocenter orientation=landscape missing=' ' nofmterr pageno=1;

/*** Site-specific location of source data ***/
```

```
libname a ( "L:\WIHS V&LASTVISIT\Access\wdbV&LASTVISIT..mdb");
libname p "\\Fu-hsing\BCU\2786 Greenblatt WIHS\SASfiles";

%include 'formats.sas';

/*** Gather relevant data from separate source files ***/

/* Visits before visit 9 did not use the calendar-based visit numbering.
We want to recode them to the calendar-based numbering of TINT.
If more than a single visit occurs during the same interval, all but the last have a missing
TINT.  We avoid using TINT later because there have been visits that occurred just
after the end of the calendar-based interval which we would lose if we were strict.
*/
proc sort data=a.vert_datebase (keep=wihsid visit tint) out=visits nodupkey;
      by wihsid descending visit;
      run;
data visits;
      set visits;
      by wihsid descending visit;
      retain visit_interval;
      if first.wihsid then visit_interval=.;
      if visit < 9 then visit2 = tint;
            else visit2 = visit;
      if not missing(visit2) then visit_interval=visit2;
      label visit2 = "Corrected visit" /* may be missing */
            visit_interval = "Visit Interval" /* never missing */;
proc sort data=visits;
      by wihsid visit;
run;
/* see a few paragraphs down for final tweaks to visits and export */

/* Spec 3.  Analytic First Visit.
   We only want to consider women who are in the hivhist file and visits
   on or after firstvis.  firstvis is usually the baseline visit (note
      there may be visits before baseline), except for sero-converters, for whom
      firstvis is the first HIV+ visit.
*/
```

```
data first (keep=wihsid firstvis)  exclude (keep=wihsid);
      set a.HIVHIST(keep=wihsid   BSVISIT POSVIS  status NEGVIS) ;
      if status = 5 then output exclude; /* sero-converter identified at death.  1 woman as of v40. */
      else do;
            firstvis=BSVISIT;
            if status=4 then firstvis=POSVIS;
            output first;
            end;
run;
proc sort data=first; by wihsid; run;
proc sort data=exclude; by wihsid; run;

data first;
      merge first (in=a) visits (in=b keep= wihsid visit visit_interval rename=(visit=firstvis));
      by wihsid firstvis;
      if a;
      firstvis = visit_interval;
      drop visit_interval;
run;

data visits;
      merge visits (in=a) exclude (in=b);
      by wihsid;
      if a and not b;
      run;
data p.visits;  ***** OUTPUT data;
      set visits;
run;

%macro cleandat(table, hasvisit=1);  * sort dataset "table", correct visit numbering, and drop certain visits ;
      %if &hasvisit %then %do;
            proc sort data=&table;
                  by wihsid visit;
            data &table;
                  merge &table (in=a) visits (in=b keep=wihsid visit visit2);
                  by wihsid visit;
                  if a and b;
```

```
                if missing(visit2) then delete;
                visit = visit2;
                drop visit2;
          proc sort data=&table;
                by wihsid visit;
          data &table;
                merge &table (in=a) first(in=b);
                by wihsid;
                /* Spec 3.  Exclude data before first analytic visit.*/
                if a and b and visit >= firstvis;
                drop firstvis;
                run;
          %end;
    %else %do;
          proc sort data=&table;
                by wihsid;
          data &table;
                merge &table (in=a) first (in=b keep=wihsid); /* using exclude allows bogus records to slip through */
                by wihsid;
                if a and b;
          run;
          %end;
%mend cleandat;
data HIVHIST;  /*** HIV status ***/
    set a.HIVHIST;
    if status in (2 4 5) then HIV=1;
    if status =1 then HIV=0;
    format HIV HIVPos. status status.;
    keep wihsid status HIV NEGVIS POSVIS;
%cleandat(HIVHIST, hasvisit=0);

data labsum; /*** CD4 count, HIV viral load (VL), & detection limit for VL ***/
    set a.labsum;
    keep wihsid visit CD4N VLOAD UD_VL ;
%cleandat(labsum);

data rab;  /* Pre-Enrollment CD4 Nadir */
```

```
      set a.rab (keep=wihsid t14nmr);
%cleandat(rab, hasvisit=0);

data vertaidsdrug; /*** Type of anti-HIV therapy ***/
      set a.vertaidsdrug;
      format THRPYV THRPY.;
      keep wihsid visit THRPYV;
%cleandat(vertaidsdrug);


data preenroll; /*** For new recruits, HIV medication history.  ***/
      set a.wihsids (keep=wihsid wihscode status02);
      label preHAART = "Received HAART before enrollment";
      format preHAART yesno.;
      if wihscode = 2 /* initial wave, despite the number */ then
            preHAART = 0;  * treat all of them as being HAART naive;
      else if status02 = 3 /* Seropositive, HAART*/ then preHAART=1;
      else if status02 in (1, 2, 4) then preHAART=0;
      else preHAART=.;
      keep wihsid preHAART;
%cleandat(preenroll, hasvisit=0);


data OBGYN; /*** Was the participant pregnant at the visit ***/
      set a.OBGYN;
       keep  wihsid visit CURPREG  ;
%cleandat(OBGYN);


data HIV_labsum;
      merge labsum HIVHIST;
      by wihsid;
run;
proc sort;
            by wihsid visit;
run;
data one;
```

```
      merge HIV_Labsum  vertaidsdrug  ;
      by wihsid visit;
run;
/* Note that first may have some ids for which we have no visits >= firstvisit.
As long as it is always used as a screen (as in cleandat) this should do no harm.
Dataset one only has valid ids and visits. */

/* Spec 6. Initial Data Definitions */
data one; merge one hivhist; by wihsid;
      if not missing(visit);
      log10VLOAD =log10(VLOAD);
      CD4=CD4n;
      if CD4n>1000 then CD4=1000; /*** Spec 7. This is to ignore differences among very high values ***/
/*** Initialize key variables: the (sometimes) filled-in versions of therapy status, VL, and CD4 ***/
      phenoThrpy=THRPYV;
      phenoVL=vload;
      phenoUndetVL=.;
      phenoCD4=CD4;
      if UD_VL=1 then phenoUndetVL=1;
      if UD_VL=2 then phenoUndetVL=0;
run;

/*** Create a shell to fill out the data set with an observation for every visit ***/
data dummy;
      set one;
      by wihsid;
      if first.wihsid;
      keep wihsid;
run;

data dummy;
      set dummy;
      do visit = -3 to (&LASTVISIT + 4);
            output;
      end;
run; * go from 4 visits before first to 4 visits after last ;
proc sort data=dummy;
```

```
      by wihsid visit;
run;
data one; /*** Spec 1, 2 and 5.  Fill in empty records for missed visits and adds some on the ends.
     Note for simplicity we always have visits back to -3, although that visit only matters for those with firstvis=1.
     ***/
      merge one dummy;
      by wihsid visit;
      drop status HIV NEGVIS POSVIS;
run;

%* step10 macro handles
Spec 8 (THRPYV) and 10 (Viral Load) interpolation.
Spec 9, No Interpolation across different therapies, still to be applied to Viral Load.
Preparation for Spec 11 (CD4) by defining variables to capture smoothing window.
The result of this is that the datset five has imputed therapy and viral load.
The first arguments are input datasts.
"last" and later arguments are output files.
This also creates intermediate files nohiv, two, three and four.;
%macro step10(one=one, first=first, HIVHIST=HIVHIST, preenroll=preenroll, OBGYN=OBGYN,
      last=last, five=five);
data &one; merge &one (in=a) &first (in=b);
      by wihsid;
      if a; * drop ids with no data;
run;  * Fill in valid firstvis for all records ;
data &one; merge &one &HIVHIST; by wihsid;
      if not missing(visit);
      run; * Fill in HIV data for all records ;

data nohiv; set &one; if hiv=.; keep wihsid; run;
data nohiv; set nohiv; by wihsid; if first.wihsid; run;
data nohiv; merge &one nohiv(in=b); by wihsid; if b; run;

data two;
      merge &one (in=a) &preenroll;
      by wihsid;
      if a;
proc sort nodupkey;
```

```
            by wihsid visit;
run;
/* Spec 4. find last real visit */
data &last; set two; if n(of VLOAD CD4 THRPYV)>0; run;
data &last; set &last; by wihsid visit; if last.wihsid; lastvisit=visit; keep wihsid lastvisit; run;
data two; merge two &last; by wihsid; run;
/*** Merge in remaining data and obtain previous 4 values of CD4, VL, and therapy data ***/
data three;
      merge two &OBGYN;
      by wihsid visit;
      lag_id=lag(wihsid);
      lag2_id=lag2(wihsid);
      lag3_id=lag3(wihsid);
      lag4_id=lag4(wihsid);

      lag_vload=lag(vload);
      lag2_vload=lag2(vload);
      lag3_vload=lag3(vload);
      lag4_vload=lag4(vload);

      lag_CD4=lag(cd4);
      lag2_CD4=lag2(cd4);
      lag3_CD4=lag3(cd4);
      lag4_CD4=lag4(cd4);

      lag_THRPYV=lag(THRPYV);
      lag2_THRPYV=lag2(THRPYV);
      lag3_THRPYV=lag3(THRPYV);
      lag4_THRPYV=lag4(THRPYV);

      lag_UD_VL=lag(UD_VL);
      lag2_UD_VL=lag2(UD_VL);
      lag3_UD_VL=lag3(UD_VL);
      lag4_UD_VL=lag4(UD_VL);

      if    wihsid^=lag_id then do; /*** Prevent use of data from other participants ***/
            lag_vload=.;
```

```
            lag_CD4=.;
            lag_THRPYV=.;
            lag_UD_VL=.;
      end;
      if    wihsid^=lag2_id then do;
            lag2_vload=.;
            lag2_CD4=.;
            lag2_THRPYV=.;
            lag2_UD_VL=.;
      end;
      if    wihsid^=lag3_id then do;
            lag3_vload=.;
            lag3_CD4=.;
            lag3_THRPYV=.;
            lag3_UD_VL=.;
      end;
      if    wihsid^=lag4_id then do;
            lag4_vload=.;
            lag4_CD4=.;
            lag4_THRPYV=.;
            lag4_UD_VL=.;
      end;
      drop lag_id lag2_id lag3_id lag4_id;
proc sort;
            by wihsid descending visit;   /*** Reverse order to obtain subsequent values below ***/
run;

/*** Obtain next 4 values of key variables ***/
data four;
      set three;
      lead_id=lag(wihsid);
      lead2_id=lag2(wihsid);
      lead3_id=lag3(wihsid);
      lead4_id=lag4(wihsid);

      lead_vload=lag(vload);
      lead2_vload=lag2(vload);
```

```
lead3_vload=lag3(vload);
lead4_vload=lag4(vload);

lead_CD4=lag(cd4);
lead2_CD4=lag2(cd4);
lead3_CD4=lag3(cd4);
lead4_CD4=lag4(cd4);

lead_THRPYV=lag(THRPYV);
lead2_THRPYV=lag2(THRPYV);
lead3_THRPYV=lag3(THRPYV);
lead4_THRPYV=lag4(THRPYV);

lead_UD_VL=lag(UD_VL);
lead2_UD_VL=lag2(UD_VL);
lead3_UD_VL=lag3(UD_VL);
lead4_UD_VL=lag4(UD_VL);

if    wihsid^=lead_id then do;
      lead_vload=.;
      lead_CD4=.;
      lead_THRPYV=.;
      lead_UD_VL=.;
end;
if    wihsid^=lead2_id then do;
      lead2_vload=.;
      lead2_CD4=.;
      lead2_THRPYV=.;
      lead2_UD_VL=.;
end;
if    wihsid^=lead3_id then do;
      lead3_vload=.;
      lead3_CD4=.;
      lead3_THRPYV=.;
      lead3_UD_VL=.;
end;
if    wihsid^=lead4_id then do;
```

```
            lead4_vload=.;
            lead4_CD4=.;
            lead4_THRPYV=.;
            lead4_UD_VL=.;
      end;
      drop lead_id lead2_id lead3_id lead4_id;
proc sort;
            by wihsid visit;  /*** Back in normal order ***/
run;

/*** Now fill in therapy and VL variables as in Specifications document ***/
data &five;
      set four;

       if THRPYV=. and lag_THRPYV>. and lead_THRPYV>. then do;               * Spec 8, one case ;
            if lag_THRPYV=lead_THRPYV then phenoThrpy=lead_THRPYV;
            else phenoThrpy=.;
       end;
        if THRPYV=. and lag_THRPYV=. and lag2_THRPYV>. and lead_THRPYV>. then do; * Spec 8, second case ;
            if lag2_THRPYV=lead_THRPYV then phenoThrpy=lead_THRPYV;
            else phenoThrpy=.;
       end;
       if THRPYV=. and lag_THRPYV>. and lead_THRPYV=. and lead2_THRPYV>. then do; * Spec 8, third case ;
            if lag_THRPYV=lead2_THRPYV then phenoThrpy=lead2_THRPYV;
            else phenoThrpy=.;
       end;

      if vload =. and lag_vload>. and lead_vload>. then do; * Spec 10.  Note that Spec 9 restriction is implementded
afterward ;

            if lag_UD_VL=1  and lead_UD_VL=1  then do; * Spec 10.a) ;
                        phenoVL=min (of lag_vload lead_vload );
                        phenoUndetVL=1;
            end;

            if lag_UD_VL=1 and  .< lead_vload<1000 and lead_UD_VL=2 then do; * Spec 10.c, one case ;
                        phenoVL= lag_vload  ;
```

```
                phenoUndetVL=1;
        end;
        if .< lag_vload<1000 and lag_UD_VL=2 and lead_UD_VL=1 then do; * Spec 10.c, other case ;
                phenoVL= lead_vload  ;
                phenoUndetVL=1;
        end;

        if (lag_UD_VL=1 and lead_vload>= 1000 and lead_UD_VL=2 )
            or
            (lag_vload >=1000 and lag_UD_VL=2 and lead_UD_VL=1 )  then do; * Spec 10.d ;
                phenoVL= . ;
                phenoUndetVL=.;
        end;

        if lag_UD_VL=2 and lead_UD_VL=2  then do;               * Spec 10.e ;
                phenoVL= sqrt((lag_vload*lead_Vload))  ;
                phenoUndetVL=0;
        end;
    end;

    if vload =. and lag_vload=. and lag2_vload>. and lead_vload>. then do; * Another case for Spec 10 ;
        if ( lag2_UD_VL=1 and lead_UD_VL=1 ) then do;  * Spec 10.a ;
                phenoVL=min (of lag2_vload lead_vload );
                phenoUndetVL=1;
        end;

        if (lag2_UD_VL=1 and .< lead_vload<1000 and lead_UD_VL=2 ) then do;  * Spec 10.c, one case ;
            phenoVL= lag2_vload  ;
            phenoUndetVL=1;
        end;
        if (.< lag2_vload<1000 and lag2_UD_VL=2  and lead_UD_VL=1 )       then do;  * Spec 10.c, other case ;
                phenoVL= lead_vload  ;
                phenoUndetVL=1;
        end;

        if (lag2_UD_VL=1 and lead_vload>= 1000 and lead_UD_VL=2 )
            or
```

```
            ( lag2_vload >=1000 and lag2_UD_VL=2 and lead_UD_VL=1 )     then do;  * Spec 10.d ;
                  phenoVL= . ;
                  phenoUndetVL=.;
      end;

      if (lag2_vload>. and lag2_UD_VL=2 and lead_vload>. and lead_UD_VL=2 ) then do;  * Spec 10.e ;
                  phenoVL= lag2_vload*(lead_Vload/lag2_Vload)**(2/3)  ;
                  phenoUndetVL=0;
      end;
end;
if vload =. and lag_vload>. and lead_vload=. and lead2_vload>. then do;  * Another case for Spec 10 ;
      if ( lag_UD_VL=1 and lead2_UD_VL=1 ) then do;  * Spec 10.a ;
                  phenoVL=min (of lag_vload lead2_vload );
                  phenoUndetVL=1;
      end;

      if (lag_UD_VL=1 and .< lead2_vload<1000 and lead2_UD_VL=2 ) then do;  * Spec 10.c, one case ;
            phenoVL= lag_vload  ;
            phenoUndetVL=1;
      end;
      if (.< lag_vload<1000 and lag_UD_VL=2 and lead2_UD_VL=1 ) then do;  * Spec 10.c, other case ;
                  phenoVL= lead2_vload  ;
                  phenoUndetVL=1;
      end;

      if (lag_UD_VL=1 and lead2_vload>= 1000 and lead2_UD_VL=2 )
            or
            (lag_vload >=1000 and lag_UD_VL=2 and lead2_UD_VL=1 )       then do;  * Spec 10.d ;
                  phenoVL= . ;
                  phenoUndetVL=.;
      end;

      if (lag_UD_VL=2 and lead2_UD_VL=2 ) then do;   * Spec 10.e ;
                  phenoVL= lag_vload*(lead2_Vload/lag_vload)**(1/3)  ;
                  phenoUndetVL=0;
      end;
end;
```

```
      if (vload=. and lag_vload=. and lag2_vload=. )
            or (vload=. and lead_vload=. and lead2_vload=.)
            or (vload=. and lag_vload=. and lead_vload=.) then do; * Spec 10.f ;
            phenoVL= . ;
            phenoUndetVL=.;
       end;
run;
%mend step10;

%* finish computation of viral load by imposing the Spec 9 restriction: no interpolation if change in therapy.
five is input
eight is primary output
produces intermediate files six and seven;
%macro step12(five=five, eight=eight);     * PB NOTE: Rename step9 ?  ;
/*** Obtain previous values of phenoThrpy ***/
data six;
      set &five;
      lag_id=lag(wihsid);
      lag2_id=lag2(wihsid);
      lag3_id=lag3(wihsid);
      lag4_id=lag4(wihsid);
      lag_phenoThrpy=lag(phenoThrpy);
      lag2_phenoThrpy=lag2(phenoThrpy);
      lag3_phenoThrpy=lag3(phenoThrpy);
      lag4_phenoThrpy=lag4(phenoThrpy);
      if    wihsid^=lag_id then do;       * Prevent use of other participants' data ;
            lag_phenoThrpy=.;
      end;
      if    wihsid^=lag2_id then do;
            lag2_phenoThrpy=.;
      end;
      if    wihsid^=lag3_id then do;
            lag3_phenoThrpy=.;
      end;
      if    wihsid^=lag4_id then do;
            lag4_phenoThrpy=.;
      end;
```

```
      drop lag_id lag2_id lag3_id lag4_id ;
proc sort;
            by wihsid descending visit;   /*** Reverse order to obtain subsequent phenoThrpy ***/
run;
/*** Obtain subsequent phenoThrpy ***/
data seven;
      set six;
      lead_id=lag(wihsid);
      lead2_id=lag2(wihsid);
      lead3_id=lag3(wihsid);
      lead4_id=lag4(wihsid);
      lead_phenoThrpy=lag(phenoThrpy);
      lead2_phenoThrpy=lag2(phenoThrpy);
      lead3_phenoThrpy=lag3(phenoThrpy);
      lead4_phenoThrpy=lag4(phenoThrpy);
      if    wihsid^=lead_id then do;       * Prevent use of other participants' data ;
            lead_phenoThrpy=.;
      end;
      if    wihsid^=lead2_id then do;
            lead2_phenoThrpy=.;
      end;
      if    wihsid^=lead3_id then do;
            lead3_phenoThrpy=.;
      end;
      if    wihsid^=lead4_id then do;
            lead4_phenoThrpy=.;
      end;
      drop lead_id lead2_id lead3_id lead4_id ;
proc sort;
            by wihsid visit;     * Put back in normal order ;
run;

/*** Apply Spec 9 restriction ***/
/*** Note: phenoThrpy cannot be missing at the current visit if not missing at previous or next visits ***/
data &eight;
      set seven;
      if vload=. then do;
```

```
            if (lag_phenoThrpy ^=lead_phenoThrpy) or (phenoThrpy^=lag_phenoThrpy) or (phenoThrpy^= lead_phenoThrpy) or
           (phenoThrpy=.) then do;
                        phenoUndetVL=.;
                        phenoVL =.;
            end;
      end;
run;
%mend step12;

%* Spec 11. Interpolate CD4, only when therapy is constant (Spec 9).
eight = input
PhenoData =output
produces intermediate datasets TmpCD4 Win1to6 Win7 pe fittedCD4 fittedCD4_Win1to6
      Win7S fittedCD4AllWin;
%macro step14(eight=eight, PhenoData=PhenoData);
/*** Find smoothing windows for Spec 11 and output data in each person's window ***/
data TmpCD4;
      set &eight;

      /*** If changes in therapy near the current visit, then no smoothing or interpolation ***/
    if (lag_phenoThrpy=phenoThrpy | visit=firstvis) & phenoThrpy>. then do; * iflevel 1, restriction specified by Spec
11.a ;
        /*** Define which visits have no change in phenoThrpy ***/
        if lag2_phenoThrpy=phenoThrpy then visminus2OK=1;
        if visminus2OK=1 & lag3_phenoThrpy=phenoThrpy then visminus3OK=1;
        if visminus3OK=1 & lag4_phenoThrpy=phenoThrpy then visminus4OK=1;
        if lead_phenoThrpy=phenoThrpy then visplus1OK=1;
        if visplus1OK=1 & lead2_phenoThrpy=phenoThrpy then visplus2OK=1;
        if visplus2OK=1 & lead3_phenoThrpy=phenoThrpy then visplus3OK=1;
        if visplus3OK=1 & lead4_phenoThrpy=phenoThrpy then visplus4OK=1;
        done=0;                          * Indicator so that lots of else statements will not be needed ;
      if cd4>. then do; * iflevel 2, Spec 11.b ;
            if lag_CD4>. & lead_CD4>. & visplus1OK=1  then do; * iflevel 3, window type 11.b.i ;
                        vnum=-1; sqrtCD4=sqrt(lag_CD4); Window=1; output;
                        vnum=0; sqrtCD4=sqrt(cd4); Window=1; output;
                        vnum=1; sqrtCD4=sqrt(lead_CD4);  Window=1;output;
                        done=1;
```

```
    end; * close iflevel 3 ;
  if done=0 & lag2_CD4>. & visminus2OK=1 & lead_CD4>. & visplus1OK=1 then do; * iflevel 3, window 11.b.ii ;
            vnum=-2; sqrtCD4=sqrt(lag2_CD4); window=1 ; output;
            vnum=0; sqrtCD4=sqrt(cd4); window=1 ;output;
            vnum=1; sqrtCD4=sqrt(lead_CD4); window=1 ;output;
            done=1;
    end; * close iflevel 3 ;
  if done=0 & lead2_CD4>. & visplus2OK=1 & lag_CD4>. then do; * iflevel 3, window 11.b.iii ;
                  vnum=-1; sqrtCD4=sqrt(lag_CD4);  Window=1; output;
                  vnum=0; sqrtCD4=sqrt(cd4);  Window=1; output;
                  vnum=2; sqrtCD4=sqrt(lead2_CD4);  Window=1; output;
                done=1;
    end; * close iflevel 3 ;
  if done=0 & lag2_CD4>. & visminus2OK=1 & lead2_CD4>. & visplus2OK=1 then do; * iflevel 3, window 11.b.iv ;
                        vnum=-2; sqrtCD4=sqrt(lag2_CD4); window=1 ; output;
                        vnum=0; sqrtCD4=sqrt(cd4); window=1 ; output;
                        vnum=2; sqrtCD4=sqrt(lead2_CD4); window=1 ; output;
                    done=1;
    end; * close iflevel 3 ;
    if done=0 & lag2_CD4>. & visminus2OK=1 & lag_CD4>. then do; * iflevel3, window 11.b.v ;
                  vnum=-2; sqrtCD4=sqrt(lag2_CD4); window=2 ; output;
                  vnum=-1; sqrtCD4=sqrt(lag_CD4); window=2 ; output;
                  vnum=0; sqrtCD4=sqrt(cd4); window=2 ; output;
            done=1;
    end; * close iflevel 3 ;
    if done=0 & lag3_CD4>. & visminus3OK=1 & lag_CD4>. then do; * iflevel3, window 11.b.vi ;
                  vnum=-3; sqrtCD4=sqrt(lag3_CD4); window=2 ; output;
                  vnum=-1; sqrtCD4=sqrt(lag_CD4); window=2 ; output;
                  vnum=0; sqrtCD4=sqrt(cd4); window=2 ;  output;
            done=1;
    end; * close iflevel 3 ;
    if done=0 & lag4_CD4>. & visminus4OK=1 & lag_CD4>. then do; * iflevel3, window 11.b.vii ;
                  vnum=-4; sqrtCD4=sqrt(lag4_CD4); window=2 ; output;
                  vnum=-1; sqrtCD4=sqrt(lag_CD4); window=2 ; output;
                  vnum=0; sqrtCD4=sqrt(cd4); window=2 ; output;
                done=1;
    end; * close iflevel 3 ;
```

```
if done=0 & lag3_CD4>. & visminus3OK=1 & lag2_CD4>. then do; * iflevel3, window 11.b.viii ;
                   vnum=-3; sqrtCD4=sqrt(lag3_CD4); window=2 ; output;
                   vnum=-2; sqrtCD4=sqrt(lag2_CD4); window=2 ;output;
                   vnum=0; sqrtCD4=sqrt(cd4); window=2 ; output;
               done=1;
end; * close iflevel 3 ;
if done=0 & lag4_CD4>. & visminus4OK=1 & lag2_CD4>. then do; * iflevel3, window 11.b.ix ;
                   vnum=-4; sqrtCD4=sqrt(lag4_CD4); window=2 ;output;
                   vnum=-2; sqrtCD4=sqrt(lag2_CD4); window=2 ;output;
                   vnum=0; sqrtCD4=sqrt(cd4); window=2 ;output;
               done=1;
end; * close iflevel 3 ;
if done=0 & lead2_CD4>. & visplus2OK=1 & lead_CD4>. then do; * iflevel3, window 11.b.x ;
        vnum=0; sqrtCD4=sqrt(cd4); window=3 ; output;
        vnum=1; sqrtCD4=sqrt(lead_CD4); window=3 ; output;
        vnum=2; sqrtCD4=sqrt(lead2_CD4);window=3 ;  output;
        done=1;
end; * close iflevel 3 ;
if done=0 & lead3_CD4>. & visplus3OK=1 & lead_CD4>. then do; * iflevel3, window 11.b.xi ;
        vnum=0; sqrtCD4=sqrt(cd4); window=3 ;  output;
        vnum=1; sqrtCD4=sqrt(lead_CD4); window=3 ; output;
        vnum=3; sqrtCD4=sqrt(lead3_CD4); window=3 ; output;
        done=1;
end; * close iflevel 3 ;
if done=0 & lead4_CD4>. & visplus4OK=1 & lead_CD4>. then do; * iflevel3, window 11.b.xii ;
        vnum=0; sqrtCD4=sqrt(cd4); window=3 ; output;
        vnum=1; sqrtCD4=sqrt(lead_CD4); window=3 ; output;
        vnum=4; sqrtCD4=sqrt(lead4_CD4); window=3 ; output;
        done=1;
end; * close iflevel 3 ;
if done=0 & lead3_CD4>. & visplus3OK=1 & lead2_CD4>. then do; * iflevel3, window 11.b.xiii ;
                    vnum=0; sqrtCD4=sqrt(cd4); window=3 ; output;
                    vnum=2; sqrtCD4=sqrt(lead2_CD4);window=3 ; output;
                    vnum=3; sqrtCD4=sqrt(lead3_CD4); window=3 ;output;
                done=1;
end; * close iflevel 3 ;
if done=0 & lead4_CD4>. & visplus4OK=1 & lead2_CD4>. then do; * iflevel3, window 11.b.xiv ;
```

```
                        vnum=0; sqrtCD4=sqrt(cd4); window=3 ;output;
                        vnum=2; sqrtCD4=sqrt(lead2_CD4); window=3 ;output;
                        vnum=4; sqrtCD4=sqrt(lead4_CD4); window=3 ;output;
                    done=1;
    end; * close iflevel 3 ;
    if done=0 then do;   * iflevel3, No valid window, so just use observed (unsmoothed) CD4 ;
                  vnum=0; sqrtCD4=sqrt(cd4); window=7; output;
                        done=1;
    end; * close iflevel 3 ;
end; * close iflevel 2, non-missing current CD4 ;
else do; * else for iflevel2, missing current CD4 count, Spec 11.c ;
     if done=0 & lag_CD4>. & lag2_CD4>. & visminus2OK=1 & lead_CD4>. & visplus1OK=1 then do; * window 11.c.i ;
                          vnum=-2; sqrtCD4=sqrt(lag2_CD4); window=4 ;output;
                          vnum=-1; sqrtCD4=sqrt(lag_CD4);window=4 ; output;
                          vnum=1; sqrtCD4=sqrt(lead_CD4); window=4 ;output;
                          done=1;
     end; * close iflevel 3 ;
     if done=0 & lag_CD4>. & lead2_CD4>. & visplus2OK=1 & lead_CD4>. then do; * window 11.c.ii) ;
                          vnum=-1; sqrtCD4=sqrt(lag_CD4); window=4 ;output;
                          vnum=1; sqrtCD4=sqrt(lead_CD4); window=4 ;output;
                          vnum=2; sqrtCD4=sqrt(lead2_CD4); window=4 ;output;
                          done=1;
     end; * close iflevel 3 ;
     if done=0 & lag_CD4>. & lag3_CD4>. & visminus3OK=1 & lead_CD4>. & visplus1OK=1 then do; * window 11.c.iii ;
                          vnum=-3; sqrtCD4=sqrt(lag3_CD4); window=4 ;output;
                          vnum=-1; sqrtCD4=sqrt(lag_CD4); window=4 ;output;
                          vnum=1; sqrtCD4=sqrt(lead_CD4); window=4 ;output;
                          done=1;
     end; * close iflevel 3 ;
     if done=0 & lag_CD4>. & lead3_CD4>. & visplus3OK=1 & lead_CD4>. then do; * window 11.c.iv ;
                          vnum=-1; sqrtCD4=sqrt(lag_CD4); window=4 ; output;
                          vnum=1; sqrtCD4=sqrt(lead_CD4); window=4 ; output;
                          vnum=3; sqrtCD4=sqrt(lead3_CD4); window=4 ; output;
                          done=1;
     end; * close iflevel 3 ;
     if done=0 & lag_CD4>. & lag4_CD4>. & visminus4OK=1 & lead_CD4>. & visplus1OK=1 then do; * window 11.c.v ;
                          vnum=-4; sqrtCD4=sqrt(lag4_CD4); window=4 ;output;
```

```
                          vnum=-1; sqrtCD4=sqrt(lag_CD4); window=4 ;output;
                          vnum=1; sqrtCD4=sqrt(lead_CD4); window=4 ;output;
                          done=1;
end; * close iflevel 3 ;
if done=0 & lag_CD4>. & lead4_CD4>. & visplus4OK=1 & lead_CD4>. then do; * window 11.c.vi ;
                          vnum=-1; sqrtCD4=sqrt(lag_CD4); window=4 ;output;
                          vnum=1; sqrtCD4=sqrt(lead_CD4); window=4 ;output;
                          vnum=4; sqrtCD4=sqrt(lead4_CD4); window=4 ;output;
                          done=1;
end; * close iflevel 3 ;
if done=0 & lag_CD4>. & lag2_CD4>. & visminus2OK=1 & lead2_CD4>. & visplus2OK=1 then do; * window 11.c.vii ;
                          vnum=-2; sqrtCD4=sqrt(lag2_CD4); window=5 ;output;
                          vnum=-1; sqrtCD4=sqrt(lag_CD4); window=5 ; output;
                          vnum=2; sqrtCD4=sqrt(lead2_CD4); window=5 ;output;
                          done=1;
end; * close iflevel 3 ;
if done=0 & lag_CD4>. & lag3_CD4>. & visminus3OK=1 & lead2_CD4>. & visplus2OK=1 then do; * window 11.c.viii ;
                          vnum=-3; sqrtCD4=sqrt(lag3_CD4); window=5 ;output;
                          vnum=-1; sqrtCD4=sqrt(lag_CD4); window=5 ;output;
                          vnum=2; sqrtCD4=sqrt(lead2_CD4); window=5 ;output;
                          done=1;
end; * close iflevel 3 ;
if done=0 & lag_CD4>. & lag4_CD4>. & visminus4OK=1 & lead2_CD4>. & visplus2OK=1 then do; * window 11.c.ix ;
                          vnum=-4; sqrtCD4=sqrt(lag4_CD4); window=5 ; output;
                          vnum=-1; sqrtCD4=sqrt(lag_CD4); window=5 ;output;
                          vnum=2; sqrtCD4=sqrt(lead2_CD4); window=5 ;output;
                          done=1;
end; * close iflevel 3 ;
if done=0 & lag2_CD4>. & lag3_CD4>. & visminus3OK=1 & lead_CD4>. & visplus1OK=1 then do; * window 11.c.x ;
                          vnum=-3; sqrtCD4=sqrt(lag3_CD4); window=5 ;output;
                          vnum=-2; sqrtCD4=sqrt(lag2_CD4); window=5 ;output;
                          vnum=1; sqrtCD4=sqrt(lead_CD4); window=5 ;output;
                          done=1;
end; * close iflevel 3 ;
if done=0 & lag2_CD4>. & lag4_CD4>. & visminus4OK=1 & lead_CD4>. & visplus1OK=1 then do; * window 11.c.xi ;
                          vnum=-4; sqrtCD4=sqrt(lag4_CD4); window=5 ;output;
                          vnum=-2; sqrtCD4=sqrt(lag2_CD4); window=5 ;output;
```

```
                          vnum=1; sqrtCD4=sqrt(lead_CD4); window=5 ;output;
                          done=1;
          end; * close iflevel 3 ;
          if done=0 & lag2_CD4>. & lag3_CD4>. & visminus3OK=1 & lead2_CD4>. & visplus2OK=1 then do; * window 11.c.xii ;
                          vnum=-3; sqrtCD4=sqrt(lag3_CD4); window=5 ; output;
                          vnum=-2; sqrtCD4=sqrt(lag2_CD4);  window=5 ; output;
                          vnum=2; sqrtCD4=sqrt(lead2_CD4);  window=5 ; output;
                          done=1;
          end; * close iflevel 3 ;
          if done=0 & lag2_CD4>. & lag4_CD4>. & visminus4OK=1 & lead2_CD4>. & visplus2OK=1 then do; * window 11.c.xiii
;
                          vnum=-4; sqrtCD4=sqrt(lag4_CD4);  window=5 ; output;
                          vnum=-2; sqrtCD4=sqrt(lag2_CD4);  window=5 ; output;
                          vnum=2; sqrtCD4=sqrt(lead2_CD4);  window=5 ; output;
                          done=1;
          end; * close iflevel 3 ;
          if done=0 & lag2_CD4>. & visminus2OK=1 & lead_CD4>. & lead2_CD4>. & visplus2OK=1 then do; * window 11.c.xiv ;
                          vnum=-2; sqrtCD4=sqrt(lag2_CD4); window=5 ;output;
                          vnum=1; sqrtCD4=sqrt(lead_CD4); window=5 ;output;
                          vnum=2; sqrtCD4=sqrt(lead2_CD4); window=5 ;output;
                          done=1;
          end; * close iflevel 3 ;
          if done=0 & lag2_CD4>. & visminus2OK=1 & lead_CD4>. & lead3_CD4>. & visplus3OK=1 then do; * window 11.c.xv ;
                          vnum=-2; sqrtCD4=sqrt(lag2_CD4); window=5 ; output;
                          vnum=1; sqrtCD4=sqrt(lead_CD4); window=5 ; output;
                          vnum=3; sqrtCD4=sqrt(lead3_CD4); window=5 ;output;
                          done=1;
          end; * close iflevel 3 ;
          if done=0 & lag2_CD4>. & visminus2OK=1 & lead_CD4>. & lead4_CD4>. & visplus4OK=1 then do; * window 11.c.xvi ;
                          vnum=-2; sqrtCD4=sqrt(lag2_CD4); window=5 ;output;
                          vnum=1; sqrtCD4=sqrt(lead_CD4); window=5 ;;
                          vnum=4; sqrtCD4=sqrt(lead4_CD4); window=5 ;output;
                          done=1;
          end; * close iflevel 3 ;
          if done=0 & lag_CD4>. & lead2_CD4>. & lead3_CD4>. & visplus3OK=1 then do; * window 11.c.xvii ;
                          vnum=-1; sqrtCD4=sqrt(lag_CD4); window=5 ;output;
                          vnum=2; sqrtCD4=sqrt(lead2_CD4); window=5 ;;
```

```
                              vnum=3; sqrtCD4=sqrt(lead3_CD4); window=5 ;output;
                              done=1;
          end; * close iflevel 3 ;
          if done=0 & lag_CD4>. & lead2_CD4>. & lead4_CD4>. & visplus4OK=1 then do; * window 11.c.xviii ;
                              vnum=-1; sqrtCD4=sqrt(lag_CD4); window=5 ;output;
                              vnum=2; sqrtCD4=sqrt(lead2_CD4); window=5 ;;
                              vnum=4; sqrtCD4=sqrt(lead4_CD4); window=5 ;output;
                              done=1;
          end; * close iflevel 3 ;
          if done=0 & lag2_CD4>. & visminus2OK=1 & lead2_CD4>. & lead3_CD4>. & visplus3OK=1 then do; * window 11.c.xix
;
                              vnum=-2; sqrtCD4=sqrt(lag2_CD4);  window=5 ;  output;
                              vnum=2; sqrtCD4=sqrt(lead2_CD4);  window=5 ; output;
                              vnum=3; sqrtCD4=sqrt(lead3_CD4);  window=5 ; output;
                              done=1;
          end; * close iflevel 3 ;
          if done=0 & lag2_CD4>. & visminus2OK=1 & lead2_CD4>. & lead4_CD4>. & visplus4OK=1 then do; * window 11.c.xx ;
                              vnum=-2; sqrtCD4=sqrt(lag2_CD4);  window=5 ; output;
                              vnum=2; sqrtCD4=sqrt(lead2_CD4);  window=5 ;output;
                              vnum=4; sqrtCD4=sqrt(lead4_CD4);  window=5 ; output;
                              done=1;
          end; * close iflevel 3 ;
          if done=0 & lag_CD4>. & lead_CD4>. & visplus1OK=1 then do; * window 11.c.xxi ;
                              vnum=-1; sqrtCD4=sqrt(lag_CD4); window=6 ; output;
                              vnum=1; sqrtCD4=sqrt(lead_CD4); window=6 ;output;
                              done=1;
          end; * close iflevel 3 ;
          if done=0 & lag_CD4>. & lead2_CD4>. & visplus2OK=1 then do; * window 11.c.xxii ;
                              vnum=-1; sqrtCD4=sqrt(lag_CD4);  window=6 ; output;
                              vnum=2; sqrtCD4=sqrt(lead2_CD4); window=6 ; output;
                              done=1;
          end; * close iflevel 3 ;
          if done=0 & lead_CD4>. & visplus1OK=1 & lag2_CD4>. & visminus2OK=1 then do; * window 11.c.xxiii ;;
                              vnum=-2; sqrtCD4=sqrt(lag2_CD4); window=6 ; output;
                              vnum=1; sqrtCD4=sqrt(lead_CD4); window=6 ; output;
                              done=1;
          end; * close iflevel 3 ;
```

```
          if done=0 & lag_CD4>. & lead2_CD4>. & visplus2OK=1 then do; * window 11.c.xxiv ;
                                     vnum=-2; sqrtCD4=sqrt(lag2_CD4);  window=6 ; output;
                                     vnum=2; sqrtCD4=sqrt(lead2_CD4);  window=6 ; output;
                                     done=1;
          end; * close iflevel 3 ;
            if done=0 then do; * No valid window for interpolation ;
                vnum=0; sqrtCD4=sqrt(cd4); Window=7; output;
            end;
        end; * end of else for iflevel2, missing current CD4 count case ;
      end; * end of iflevel1, no change in therapy near current visit ;
      else do;  * else for iflevel 1, change in therapy near current visit ;
                         vnum=0; sqrtCD4=sqrt(cd4); Window=7; output;
      end; * close else for iflevel 1 ;
      keep wihsid visit vnum sqrtCD4 window;
run;

options notes;
data Win1To6 Win7;
      set tmpcd4;
      if window <=6 then output Win1To6 ;
      if window=7 then output win7;
run;

proc sort data=Win1To6;
by wihsid visit;
run;

/* Precautionary statement in case a working data set is left over from runs of other programs, or previous run of this
one */
proc datasets; delete pe;
run; quit;
*options nonotes; *disabled so warning messages are accompanied by group identifying info;
ods listing close;
ods noresults;
/*** Apply linear regression to data in window for each visit ***/
/* The following regression produces many warnings about the range of variation in sqrtCD4 being
too small.  I have inspected a couple of cases with a small range of values.
```

```
The parameter estimates are fine, with the estimated slope being 0 or nearly so.

This code is very slow when I run it interactively using remote desktop; it generates
lots of plot diagnostics.  In batch mode the run time is trivial.  Judicious use of ODS
or other options could suppress the interactive output.

Ross Boylan 2015-06-23. */
proc reg data=Win1To6;
 by wihsid visit;
  model sqrtCD4 = vnum;
  ods output  ParameterEstimates=pe;
run;
quit;
options notes;
ods listing;

/*** Intercept is the smoothed/interpolated value of sqrtCD4 for each visit ***/
data fittedCD4;
      set pe;
      if variable='Intercept';
      fittedRootCD4=estimate;
proc sort nodupkey;
            by wihsid visit;
run;
/*** Merge in the variable "window" ***/
data fittedCD4_Win1To6;
      merge Win1To6 (keep=wihsid visit window) fittedCD4(keep=wihsid visit fittedRootCD4);
      by wihsid visit;
      if first.wihsid or first.visit;
run;

/*** The visits with no smoothing/interpolation ***/
proc sort data=win7 nodupkey out=Win7S;
by wihsid visit;
run;

/*** Combine all fitted data ***/
```

```
data  fittedCD4AllWin ;
      set fittedCD4_Win1To6 Win7S (keep=wihsid visit window sqrtCD4 rename=(sqrtCD4 =fittedRootCD4));
proc sort;
            by wihsid visit;
run;

/*** Add smoothed/interpolated CD4 values to the phenotyping data set ***/
data  &PhenoData;
      merge eight  fittedCD4AllWin ;
      by wihsid visit;
      phenoCD4=fittedRootCD4*fittedRootCD4;
run;
%mend step14;
** RB reviewed to here.  Spec 1-11 have been referenced.

%* The macro limits the range of visits per Spec 2-5;
%macro step20;
data phenoData; set phenoData; if visit>=firstvis & visit<=lastvisit; run;

data pheno; set phenoData; run;

proc sort; by wihsid visit; run;
%mend step20;

%* Main phenotype definitions.
inputs: pheno (which is modified and thus an output too)
outputs: out, ordinarily a persistent dataset
No itermediate datasets;
%macro step22(pheno=pheno, out=p.pheno_6_21_12);
data &pheno; set &pheno; by wihsid visit;
  retain phenoHAART phenoHAART2; * # of consecutive visits on HAART -- Spec 12, 13 ;
  * phenoHAART is the count of consecutive visits on HAART ;
  * phenoHAART2 is the minimum number of possible consecutive visits on HAART when it is not known exactly ;
  if first.wihsid & phenoThrpy=3 then do;
      phenoHAART=1;
      phenoHAART2=1;
      end;
```

```
  if first.wihsid & phenoThrpy=3 & (preHAART=1 or missing(preHAART)) then do;
       * May have already been on HAART ;
       phenoHAART=. /*Spec 12g + 12h1*/; phenoHAART2=1 /*Spec 13f in part*/;
       end;
  if not(first.wihsid) & phenoThrpy=3 then phenoHAART=phenoHAART+1; *Spec 12b (since phenoHAART=0 if previous phenoTrpy
            is <3 and not missing), 12c, 12d, 12f;
  if phenoThrpy=. then do; phenoHAART=. /* Spec 12e */; phenoHAART2=. /*Spec 13e */; end;
  if .<phenoThrpy<3 then do; phenoHAART=0 /* Spec 12a */; phenoHAART2=0 /* Spec 13a */; end;
  if phenoHAART>. then phenoHAART2=.;
  if phenoHAART=. then do;
    if phenoHAART2>.  & phenoThrpy=3 & not(first.wihsid) then phenoHAART2=phenoHAART2+1;
    if phenoHAART2=. & phenoThrpy=3 then phenoHAART2=1;
  end;
  if phenoHAART2>4 & phenoHAART=. then phenoHAART=phenoHAART2;
     * End of Spec 12, 13 definitions ;
  /*** Define Window for Spec 17 definitions, long-term off therapy ***/
  prevVisits=visit-firstvis;
  if prevVisits<7 and preHAART=0 then window=prevVisits;
  else window=6;

data &pheno; set &pheno; by wihsid visit; *** Define CD4 nadir -- Spec 14 ;
  retain phenoCD4nadir;
  if first.wihsid then phenoCD4nadir=phenoCD4;
  if phenoCD4nadir=. and phenoCD4>. then phenoCD4nadir=phenoCD4;
  if .<phenoCD4<phenoCD4nadir then phenoCD4nadir=phenoCD4;
data &pheno (drop=t14nmr delta) changed;
      merge &pheno (in=a) rab(in=b where=(t14nmr>0));
      by wihsid;
      if a;
      if b and t14nmr< phenoCD4Nadir then do;
            delta=phenoCD4nadir-t14nmr;
            output changed;
            phenoCD4Nadir=t14nmr;
            end;
      output &pheno;
run;
proc univariate data=changed;
```

```
      title "lowered CD4 nadirs";
      var delta;
      id wihsid visit;
      run;
proc summary data=changed;
      by wihsid;
      output out=changedN n(delta)=N;
proc freq data=changedN;
      title "Number of visits with changed CD4 nadir  per id";
      table N / missing;
      run;
/*** Obtain previous values of variables to be used for phenotyping ***/
data &pheno; set &pheno; by wihsid visit;
  lag_id = lag(wihsid);
  lag2_id = lag2(wihsid);
  lag3_id = lag3(wihsid);
  lag4_id = lag4(wihsid);
  lag5_id = lag5(wihsid);
  lag6_id = lag6(wihsid);
  phenoUndetVLPrev1=lag(phenoUndetVL);
  phenoUndetVLPrev2=lag2(phenoUndetVL);
  phenoUndetVLPrev3=lag3(phenoUndetVL);
  phenoUndetVLPrev4=lag4(phenoUndetVL);
  phenoUndetVLPrev5=lag5(phenoUndetVL);
  phenoUndetVLPrev6=lag6(phenoUndetVL);
  phenoVLPrev1=lag(phenoVL);
  phenoVLPrev2=lag2(phenoVL);
  phenoVLPrev3=lag3(phenoVL);
  phenoVLPrev4=lag4(phenoVL);
  phenoVLPrev5=lag5(phenoVL);
  phenoVLPrev6=lag6(phenoVL);
  phenoCD4Prev1=lag(phenoCD4);
  phenoCD4Prev2=lag2(phenoCD4);
  phenoCD4Prev3=lag3(phenoCD4);
  phenoCD4Prev4=lag4(phenoCD4);
  phenoCD4Prev5=lag5(phenoCD4);
  phenoCD4Prev6=lag6(phenoCD4);
```

```
phenoThrpyPrev1=lag(phenoThrpy);
phenoThrpyPrev2=lag2(phenoThrpy);
phenoThrpyPrev3=lag3(phenoThrpy);
phenoThrpyPrev4=lag4(phenoThrpy);
phenoThrpyPrev5=lag5(phenoThrpy);
phenoThrpyPrev6=lag6(phenoThrpy);
if wihsid^=lag_id then do;  /*** Prevent use of values from other women ***/
  phenoUndetVLPrev1=.;
    phenoVLPrev1=.;
    phenoCD4Prev1=.;
    phenoThrpyPrev1=.;
end;
if wihsid^=lag2_id then do;  /*** Prevent use of values from other women ***/
  phenoUndetVLPrev2=.;
    phenoVLPrev2=.;
    phenoCD4Prev2=.;
    phenoThrpyPrev2=.;
end;
if wihsid^=lag3_id then do;  /*** Prevent use of values from other women ***/
  phenoUndetVLPrev3=.;
    phenoVLPrev3=.;
    phenoCD4Prev3=.;
    phenoThrpyPrev3=.;
end;
if wihsid^=lag4_id then do;  /*** Prevent use of values from other women ***/
  phenoUndetVLPrev4=.;
    phenoVLPrev4=.;
    phenoCD4Prev4=.;
    phenoThrpyPrev4=.;
end;
if wihsid^=lag5_id then do;  /*** Prevent use of values from other women ***/
  phenoUndetVLPrev5=.;
    phenoVLPrev5=.;
    phenoCD4Prev5=.;
    phenoThrpyPrev5=.;
end;
if wihsid^=lag6_id then do;  /*** Prevent use of values from other women ***/
```

```
    phenoUndetVLPrev6=.;
      phenoVLPrev6=.;
      phenoCD4Prev6=.;
      phenoThrpyPrev6=.;
  end;
run;
/***   Define variables for use in Spec 18 definitons, short-term off-therapy
     onTxCD4avg = "average of last two on-Tx phenoCD4" in Spec 18 specifications
     phenoOffHAART  = number of consecutive visits on HAART, if known exactly (Spec 15)   ***/
data &pheno; set &pheno; by wihsid visit;
  retain onTxCD4avg phenoOffHAART;
  * Spec 15;
  if first.wihsid | phenoThrpy>2 then onTxCD4avg=.;
  if phenoThrpy in (0, 1, 2) & phenoThrpyPrev1>2 & phenoThrpyPrev2>2 then /* Just off Tx after being on for previous 2
visits  */
            onTxCD4avg=mean(of phenoCD4Prev1 phenoCD4Prev2); * average of non-missing values from previous 2 visits ;
  else if phenoThrpy in (0, 1, 2) & phenoThrpyPrev1>2 then onTxCD4avg=phenoCD4Prev1; * Similar case but only on Tx for
1 visit ;
  * Spec 16;
  if phenoThrpy = 3 then phenoOffHAART = 0; *16a;
  else if missing(phenoThrpy) and visit > 4 then phenoOffHAART=.; *16b;
  else if first.wihsid then phenoOffHAART=.; *16c;
  /* The following if clause if probably unnecessary, but it is safer, more explicit,
  and more obviously in line with the spec to keep it.  the phenoOffHAART+1 statement is necessary.  RB */
  else if phenoThrpy in (0, 1, 2) | (missing(phenoThrpy) and visit<=4) then
             /* Note that sum statements like "phenoOffHAART+1;" ignore missing and so set
                  the variable to 1 when it should be missing,*/
            if not missing(phenoOffHAART) then phenoOffHAART=phenoOffHAART+1; *16c;
run;
/*** define L group variables, Spec 17 ***/
data &pheno; set &pheno;
      by wihsid visit;
  sumUndet=phenoUndetVL;  * initialize number of undetectable VL in window ;
  array undetPrev {6} phenoUndetVLPrev1-phenoUndetVLPrev6;
  do i=1 to window; sumUndet=sumUndet+undetPrev[i]; end; * count undetectable VL in window ;
  sumThrpy=phenoThrpy;  * This sum will only be used to check for no HAART in window ;
  array ThrpyPrev {6} phenoThrpyPrev1-phenoThrpyPrev6;
```

```
  do i=1 to window;
      if missing(ThrpyPrev[i]) then do;
            /* We might know for sure there had been therapy from other values in the window.
          But later on we will treat missing sumThrpy and sumThrpy>0 as equivalent.
          Only sumThrpy=0 conclusively shows absence of HAART from the entire window. */
            sumThrpy=.;
            leave;
            end;
      else sumThrpy=sumThrpy+(ThrpyPrev[i]>2);
      end;
  maxVL=phenoVL*(1-phenoUndetVL);                        * Maximum viral load in window ;
  if .<phenoVL*(1-phenoUndetVL)<=2000 then VL2000num=0;  * Number of viral loads >2000 in window ;
  if phenoVL*(1-phenoUndetVL)>2000 then VL2000num=1;
  array VLPrev {6} phenoVLPrev1-phenoVLPrev6;
  do i=1 to window;
    VLup=VLprev[i]*(1-undetPrev[i]);
      if VLup>maxVL & maxVL>. then maxVL=VLup;
      if VLup=. then maxVL=.;
      if VLup>2000 then VL2000num=VL2000num+1;
      if VLup=. & VL2000num<2 then VL2000num=.;
  end;
/*** define S group variables, for Spec 18 ***/
  if .<phenoOffHAART<7 then do;
      Sundet=phenoUndetVL;
      if phenoOffHAART>1 then do i=1 to (phenoOffHAART-1); Sundet=Sundet+undetPrev[i]; end;
    SmaxVL=phenoVL*(1-phenoUndetVL);
    if .<phenoVL*(1-phenoUndetVL)<=2000 then SVL2000num=0;
    if phenoVL*(1-phenoUndetVL)>2000 then SVL2000num=1;
    if phenoOffHAART>1 then do i=1 to (phenoOffHAART-1);
      VLup=VLprev[i]*(1-undetPrev[i]);
      if VLup>SmaxVL & SmaxVL>. then SmaxVL=VLup;
      if VLup=. then SmaxVL=.;
        if VLup>2000 then SVL2000num=SVL2000num+1;
        if VLup=. & SVL2000num<2 then SVL2000num=.;
    end;
  end;
run;
```

```
data &out; set &pheno; length pheno $ 3;
*** Define L groups, Spec 17 ;
  if Window>1 & sumThrpy=0 & (sumUndet=Window+1 | (sumUndet=Window & maxVL<=1000)) then do;  * Spec 17.a ;
    if phenoCD4nadir>=500 then pheno="L1a";
    else if phenoCD4nadir>. then pheno="L1b";
    else pheno="L1x";
  end;
  else if Window>1 & sumThrpy=0 & .<VL2000num<2 then do;  * Spec 17.b ;
    if phenoCD4nadir>=500 then pheno="L2a";
      else if phenoCD4nadir>. then pheno="L2b";
      else pheno="L2x";
  end;
  else if Window>1 & sumThrpy=0 & VL2000num>1 then do;  * Spec 17.c ;
    if phenoCD4nadir>=500 then pheno="L3a";
      else if phenoCD4nadir>. then pheno="L3b";
      else pheno="L3x";
  end;
*** Define S groups, Spec 18;
  if pheno="" & phenoOffHAART>1 & (Sundet=phenoOffHAART | (Sundet=(phenoOffHAART-1) & SmaxVL<=1000)) then do;  * Spec
18.a ;
    if phenoCD4>=onTxCD4avg*(0.9**phenoOffHAART)>. then pheno="S1a";
      else if phenoCD4>. & onTxCD4avg>. then pheno="S1b";
      else pheno="S1x";
  end;
  if pheno="" & phenoOffHAART=1 & (Sundet=phenoOffHAART) then do; * Spec 18.a for first off tx visit ;
    if phenoCD4>=onTxCD4avg*(0.9**phenoOffHAART)>. then pheno="S1a";
      else if phenoCD4>. & onTxCD4avg>. then pheno="S1b";
      else pheno="S1x";
  end;
  if pheno="" & phenoOffHAART>1 & (.<SVL2000num<2) then do;  * Spec 18.b ;
    if phenoCD4>=onTxCD4avg*(0.9**phenoOffHAART)>. then pheno="S2a";
      else if phenoCD4>. & onTxCD4avg>. then pheno="S2b";
      else pheno="S2x";
  end;
  if pheno="" & phenoOffHAART>1 & SVL2000num>1 then do;  * Spec 18.c ;
    if phenoCD4>=onTxCD4avg*(0.9**phenoOffHAART)>. then pheno="S3a";
      else if phenoCD4>. & onTxCD4avg>. then pheno="S3b";
```

```
      else pheno="S3x";
    end;
  *** Define H groups, Specs 19-22 ;
    if phenoHAART=1 then do;
      pheno="H0x"; * Spec 21.a -- Default for first HAART visit unless already good ;
      respcut=phenoCD4nadir+50;
        if respcut>350 then respcut=350;
        if phenoUndetVL=1 & phenoCD4>=respcut & respcut>. then pheno="H1a"; * Spec 19.a ;
        else if phenoUndetVL=1 then pheno="H1x";  * Spec 19.c ;
    end;
    if phenoHAART>1 & phenoUndetVL=1 then do;
      respcut=phenoCD4nadir+50*(phenoHAART-1);
        if respcut>phenoCD4nadir+200 then respcut=phenoCD4nadir+200;
        if respcut>350 then respcut=350;
        if phenoCD4>=respcut & respcut>. then pheno="H1a";  * Spec 19.a ;
        else if phenoCD4>. & respcut>. then pheno="H1b";  * Spec 19.b ;
        else if phenoCD4=. then pheno="H1x";  * Spec 19.c ;
        else if phenoCD4>=350 then pheno="H1a"; * Spec 19.a ;
        else if respcut=. then pheno="H1x";  * Spec 19.c ;
    end;
    if phenoHAART=. & phenoHAART2>. & phenoUndetVL=1 then do;
      respcut2=phenoCD4nadir+50*(phenoHAART2-1);
        if respcut2>phenoCD4nadir+200 then respcut2=phenoCD4nadir+200;
        if respcut2>350 then respcut=350;
      if phenoCD4>=350 then pheno="H1a";  * Spec 19.a, additional case ;
      else if phenoCD4>=phenoCD4nadir+200>. then pheno="H1a";  * Spec 19.a, additional case ;
        else if .<phenoCD4<respcut2 then pheno="H1b";  * Spec 19.b, additional case ;
      else if phenoUndetVL=1 & phenoHAART=. then pheno="D1x";  * Spec 20 ;
    end;
    if phenoHAART>1 & phenoUndetVL=0 then do;  * Spec 21, cases where not first HAART visit ;
      respcut=phenoCD4nadir+50*(phenoHAART-1);
        if respcut>phenoCD4nadir+200 then respcut=phenoCD4nadir+200;
        if respcut>350 then respcut=350;
        if phenoCD4>=respcut & respcut>. then pheno="H3a";  * Spec 21.b ;
        else if phenoCD4>. & respcut>. then pheno="H3b";  * Spec 21.c ;
        else if respcut=. & phenoCD4>=350 then pheno="H3a";  * Spec 21.b, missing respcut case ;
        else pheno="H3x";  * Spec 21.d ;
```

```
  end;
  if phenoHAART=. & phenoHAART2>. & phenoUndetVL=0 then do;
    respcut2=phenoCD4nadir+50*(phenoHAART2-1);
      if respcut2>phenoCD4nadir+200 then respcut2=phenoCD4nadir+200;
      if respcut2>350 then respcut=350;
    if phenoCD4>=350 then pheno="H3a";  * Spec 21.b, additional case ;
    else if phenoCD4>=phenoCD4nadir+200>. then pheno="H3a";  * Spec 21.b, additional case ;
      else if .<phenoCD4<respcut2 then pheno="H3b";  * Spec 21.c, additional case ;
    else pheno="D3x";  * Spec 22 ;
  end;
  if HIV=0 & visit=<lastvisit then pheno="N";  * Spec 23 ;
  if pheno="" & visit<=lastvisit then pheno="U";  * Spec 24 ;
run;
%mend step22;

%let test=0;
%macro do_all; *the following conditional statement must be inside a macro;
      %if &test = 0 %then %do;
            %step10;
            %step12;
            %step14;
            %step20;
            %step22(out=p.pheno_20160410);
            %end;
      %else %do;
            %step10;
            /* record data for diagnostic testing.
            The intent is to use the interpolated values of phenoThrpy and
            the raw values of CD4 and VL. */
            data p.step5;
                  set five;
                  run;
            proc import datafile="..\EXCEL\benchmark.csv" out=mytest;
            proc sort data=mytest (keep=wihsid) out=mytestids nodupkey;
                  by wihsid;
            data computed;
                  merge five (keep=wihsid visit thrpyv phenothrpy) mytestids (in=b);
```

```
            by wihsid;
            if b;
      *proc export data=computed outfile="computed.csv" replace;
      proc compare base=mytest compare=computed;
            id wihsid visit;
            var thrpyv phenothrpy;
            run;
      %end;
%mend do_all;
*invoke the code;
%do_all;
```
